# Reciprocal regulation between RACGAP1 and AR contributes to endocrine therapy resistance in prostate cancer

**DOI:** 10.1186/s12964-024-01703-w

**Published:** 2024-06-19

**Authors:** Jiajia Wang, Hui Liu, Zeyuan Yu, Qianqian Zhou, Feifei Sun, Jingying Han, Lin Gao, Baokai Dou, Hanwen Zhang, Jiawei Fu, Wenqiao Jia, Weiwen Chen, Jing Hu, Bo Han

**Affiliations:** 1 The Key Laboratory of Experimental Teratology, Department of Pathology, School of Basic Medical Sciences, Ministry of Education, Shandong University, Jinan, 250012 Shandong China; 2 Department of Pathology, Qilu Hospital, Shandong University, Jinan, 250012 China; 3 Michigan Center for Translational Pathology, University of Michigan, Ann Arbor, MI USA; 4 Department of Biochemistry and Molecular Biology, School of Basic Medical Sciences, Shandong University, Jinan, 250012 Shandong China; 5 Department of Pharmacy, Shandong Provincial Hospital Affiliated to Shandong First Medical University, Jinan, 250021 Shandong China

**Keywords:** CRPC, Endocrine therapy resistance, RACGAP1, AR, AR-V7, MDM2

## Abstract

**Background:**

Endocrine resistance driven by sustained activation of androgen receptor (AR) signaling pathway in advanced prostate cancer (PCa) is fatal. Characterization of mechanisms underlying aberrant AR pathway activation to search for potential therapeutic strategy is particularly important. Rac GTPase-activating protein 1 (RACGAP1) is one of the specific GTPase-activating proteins. As a novel tumor proto-oncogene, overexpression of RACGAP1 was related to the occurrence of various tumors.

**Methods:**

Bioinformatics methods were used to analyze the relationship of expression level between RACGAP1 and AR as well as AR pathway activation. qRT-PCR and western blotting assays were performed to assess the expression of AR/AR-V7 and RACGAP1 in PCa cells. Immunoprecipitation and immunofluorescence experiments were conducted to detect the interaction and co-localization between RACGAP1 and AR/AR-V7. Gain- and loss-of-function analyses were conducted to investigate the biological roles of RACGAP1 in PCa cells, using MTS and colony formation assays. In vivo experiments were conducted to evaluate the effect of RACGAP1 inhibition on the tumor growth.

**Results:**

RACGAP1 was a gene activated by AR, which was markedly upregulated in PCa patients with CRPC and enzalutamide resistance. AR transcriptionally activated RACGAP1 expression by binding to its promoter region. Reciprocally, nuclear RACGAP1 bound to the N-terminal domain (NTD) of both AR and AR-V7, blocking their interaction with the E3 ubiquitin ligase MDM2. Consequently, this prevented the degradation of AR/AR-V7 in a ubiquitin-proteasome-dependent pathway. Notably, the positive feedback loop between RACGAP1 and AR/AR-V7 contributed to endocrine therapy resistance of CRPC. Combination of enzalutamide and in vivo cholesterol-conjugated RIG-I siRNA drugs targeting RACGAP1 induced potent inhibition of xenograft tumor growth of PCa.

**Conclusion:**

In summary, our results reveal that reciprocal regulation between RACGAP1 and AR/AR-V7 contributes to the endocrine resistance in PCa. These findings highlight the therapeutic potential of combined RACGAP1 inhibition and enzalutamide in treatment of advanced PCa.

**Supplementary Information:**

The online version contains supplementary material available at 10.1186/s12964-024-01703-w.

## Introduction

According to the latest statistics, prostate cancer (PCa) accounts for 29% of all new cases of male malignant tumors, ranking the first in the incidence and second in cancer-related mortality of male malignance [1]. Due to the critical role of the androgen receptor (AR) in carcinogenesis and progression of cancer, targeting AR signaling has become mainstream approach for PCa therapy [2]. Androgen deprivation therapy (ADT) and the clinical application of second-generation potent AR inhibitors have greatly benefited PCa patients. However, acquired resistance inevitably occurs due to continuous activation of the AR pathway [3–6]. Investigation of the molecular mechanisms underlying the sustained activation of AR pathway in AR-targeted therapy is critical.

Several mechanisms have been suggested for AR pathway activation, such as androgen biosynthesis within tumors, amplification or overexpression of the AR, AR splicing variants, crosstalk between AR pathway and other pathways [7]. AR-V7, the most common splice variant of AR [8], lacking the ligand-binding domain (LBD), continuously stimulates the AR transcriptional program or activates AR target genes in a ligand-independent manner [9]. AR-V7 is detectable in the circulating tumor cells of 29–46% metastasis castration-resistant prostate cancer (CRPC) patients [10], linking resistance and inferior clinical outcomes with androgen targeted therapy [11, 12]. We and others found that, under enzalutamide treatment, the reduced degradation of AR and/or AR-V7 confers resistance to enzalutamide [13–15]. The development of resistance occurs when certain proteins, KIF15, DBC1 or lncRNAs, hijack the AR ubiquitin-proteasome degradation system, disrupting the balance of ubiquitination and deubiquitination of AR.

RACGAP1 is a member of the Rho GTPase Activating Protein family. RACGAP1 catalyzes intrinsic Rho GTPase, facilitating the translation between the GDP-bound inactive state and GTP-bound active state in various cellular processes [16]. RACGAP1 is well documented as the components of central spindlin complex in cytokinesis [17–23]. Subcellular localization of RACGAP1 regulates cell dissociation though microtubule dynamics [24], suggesting that the cytoplasmic and nuclear components of RACGAP1 may have distinct roles in cellular processes. Several studies have reported that RACGAP1 is upregulated in various malignancies, exerting pro-oncogenic effects independent of its GTPase activity [25–28]. In cervical cancer, RACGAP1 could modulate the expression and phosphorylation levels of c-Jun, a component of AP-1, through distinct pathways involving miR-192 and p-JNK [25]. Song et al. reported that upregulated RACGAP1 by E2F1 resulted in poorer prognosis in PCa patients [29].

In this study, we unveiled the upregulation of RACGAP1 in CRPC patients and established its important role in maintaining the AR signaling pathway in PCa using In vitro and In vivo experiments. Importantly, the combination of RACGAP1 inhibition and enzalutamide significantly inhibited tumor progression, suggesting a promising therapeutic strategy for overcoming endocrine treatment resistance in PCa.

## Materials and methods

### Cell culture and reagents

C4-2B, LNCaP, 22RV1, PC3 and HEK293T (CRL-3216) cells were purchased from the American Type Culture Collection (ATCC) (Rockville, MD, USA) and cultured as recommended by ATCC. All cell lines utilized in this study were verified to be free of mycoplasma infection and underwent authentication. Enzalutamide-resistant C4-2B cells were previously constructed in our laboratory [15]. Enzalutamide was purchased from Med Chem Express.

### Plasmids and transfection

RACGAP1 (Gene ID: 29,127; vector: p-Enter), AR (Gene ID: 367; vector: p-Enter), AR-V7 (Gene ID: 367, NM_001348061.1; vector: pcDNA3.1), nuclear localization signal (NLS) mutant RACGAP1 (Gene ID: 29,127; vector: PCMN-6×His-C) and MDM2 (Gene ID: 4193; vector: pcDNA3.1) plasmids were constructed by Biosune Biotech (Shanghai, China). Lipofectamine 3000 (Invitrogen, CA) was used in transfection in this study according to the manufacturers’ guidelines. shRNA targeting RACGAP1 were as follows: (shNC: TTCTCCGAACGTGTCACGTTT; shRACGAP1: GCUGAAGCAUGCACGUAAU). A lists of siRNA sequences were provided in Supplementary Table S1.

### Patients and tissue specimens

A total of three tissue microarrays were constructed representing 132 clinically localized PCa patients who underwent radical prostatectomy, and 25 PCa patients with CRPC treated by transurethral resection of the prostate between 2012 and 2015 at Qilu Hospital of Shandong University (Jinan, China). The clinical information of 132 localized PCa patients was shown in Supplementary Table S2. Two cores (0.6 mm in diameter) were taken from each representative tumor focus and the morphology was confirmed by two pathologists (J.H. and B.H.). This study was conducted following the International Ethical Guidelines for Biomedical Research Involving Human Subjects. This study protocol was approved by Shandong University Medical Research Ethics Committee according to the Declaration of Helsinki (Document No. ECSBMSSDU2021-1-61).

### Immunohistochemistry (IHC)

IHC was performed on 5-µm-thick formalin-fixed, paraffin-embedded tissue sections. The slides were dewaxed, hydrated, and went through antigen retrieving with EDTA 9.0, then incubated with the specific primary antibodies overnight at 4℃. Slides were washed and incubated with secondary antibodies for one hour at room temperature. Slides were assessed after staining with DAB and hematoxylin reagent. Primary antibodies utilized in this study were anti-RACGAP1 (ab2270, Abcam, Burlingame, USA), anti-AR (5153, Cell Signaling Technology, USA), anti-PSA (10679-1-Ap, Proteintech, China) and Ki67 (ZA-0502, Zsbio, China).

### RNA extraction and quantitative real-time PCR (qRT-PCR)

Total RNA was isolated from cells using TRIzol reagents (Invitrogen, Carlsbad, CA) following the manufacturers’ instructions. The ReverTra Ace qPCR Kit was used to reverse transcribe RNA into cDNA. The SYBR Green PCR kit (Toybo, Japan) was used to test the mRNA levels according to gene-specific primers. Primer sequences used were listed in the Supplementary Table S3.

### Western blotting and immunoprecipitation

Western blotting and immunoprecipitation assays were carried out as described previously [30]. Primary antibodies used in western blotting were anti-RACGAP1 (NBP1-33455, NOVUS, Colorado, USA), anti-AR (5153, Cell Signaling Technology, Massachusetts, USA), anti-AR-V7 (19,672, Cell Signaling Technology, Massachusetts, USA), anti-MDM2 (86,934, Cell Signaling Technology, Massachusetts, USA), anti-MYC-tag (600,032-Ig, Proteintech, China), anti-H3 (10265-1-AP, Proteintech, China), anti-HA-tag (66006-2-Ig, Proteintech, China), anti-PSA (10679-1-AP, Proteintech, China), anti-TMPRSS2 (CY8435, Abways, China), anti-His-tag (66005-1-Ig, Proteintech, China), anti-Flag-tag (66008-4-Ig, Proteintech, China) and anti-GAPDH (10494-1-AP, Proteintech, China).

### Tumor xenografts

All experiments procedures involving animals were approved by the Animal Care Committee of Shandong University (Document No. ECSBMSSDU2021-2-126). Male BALB/c nude mice, four-week-old, were purchased from Vital River Experiment Animal Technology (Beijing, China). For the effect of RACGAP1 on enzalutamide resistant (ENZR) tumor growth, 8.0 × 10^6^ C4-2B-ENZR cells expressing a control shRNA(shNC) or shRACGAP1 were mixed with Matrigel (1:1) (BD, Biosciences) and injected subcutaneously into the flanks of mice (*n* = 6/group). The mice were then surgically castrated, and treated with enzalutamide (10 mg/kg, p.o.) three times per week. To evaluate the combination of RACGAP1 inhibition and enzalutamide on ENZR tumors, 8.0 × 10^6^ C4-2B-ENZR cells were inoculated subcutaneously into the mice with 50% Matrigel. The mice were surgically castrated when xenograft tumors diameter reached a palpable stage (3 ~ 5 mm). When tumors grew back to the pre-castration size, mice were randomly divided into four groups (*n* = 6/group) and treated with vehicle control, enzalutamide (10 mg/kg, p.o.), siRACGAP1 (3 nmol) or enzalutamide (10 mg/kg, p.o.) plus siRACGAP1 (3 nmol). RACGAP1 inhibition was conducted using In vivo cholesterol-conjugated RIG-I siRNA (RiboBio, China). The mice received treatment with siRACGAP1 (3 nmol) or its negative siRNA control (RiboBio) in 50µl saline buffer every 3 days by injecting into the tumor mass directly. Next, tumor size was monitored twice a week using calipers, and the tumor volume was calculated using the formula: tumor volume = length× width^2^ × 0.5. The animals were sacrificed after 5 weeks of treatment, collecting and weighing the tumors. Tumor tissues were embedded in paraffin and conducted with IHC staining. The experimental protocols were performed following the Ethical Animal Care and Use Committee of Shandong University (Document No. ECSBMSSDU2021-2-126).

### RNA sequencing (RNA-seq) and bioinformatics analysis

RNA-seq data from the GEO (https://www.ncbi.nlm.nih.gov/geo/) database (GSE74367, GSE2443, GSE29079), GEPIA (http://gepia.cancer-pku.cn/) and MSKCC (https://www.cbioportal.org/) database were used to analyze the relationship of mRNA levels between RACGAP1 and AR. RNA-seq data from GEO database (GSE32269, GSE35988, GSE151083, GSE103449, GSE77930, GSE3325, GSE74367, GSE150807, GSE104935, GSE149433, GSE27616, GSE21034 and GSE38241) were used to compare the expression of RACGAP1 in different groups. RNA-Seq (Novogene Technology, Beijing) was performed to analysis the mRNA expression profiles between control (NC) and RACAGP1 knockdown (siRACGAP1) in 22RV1 cells. The expressed genes were analyzed for enrichment of biological themes using Gene Set Enrichment Analysis (GSEA). The Androgen-induced genes signature, Androgen-repressed gene signature and AR-V7-activated gene signature were obtained from Cato et al. [31]. The upstream TFs targeting RACGAP1 was predicted by Motif-based sequence analysis tools JASPAR (http://jaspar.genereg.net.), GTRD (https://gtrd20-06.biouml.org/) and PROMO (https://alggen.lsi.upc.es/).

### Statistical analysis

Statistical analyses in this study were conducted using GraphPad Prism 9. Data were shown as the mean ± SD from at least three independent experiments. The two-tailed unpaired t test was used to determine the difference between two groups. The significance of correlations was assessed using either Chi-Square Test. The ANOVA analysis was performed to assess tumor growth. A *p*-value less than 0.05 was used to indicate statistical significance.

## Results

### RACGAP1 is androgen responsive and upregulated in advanced PCa

**Fig. 1:**
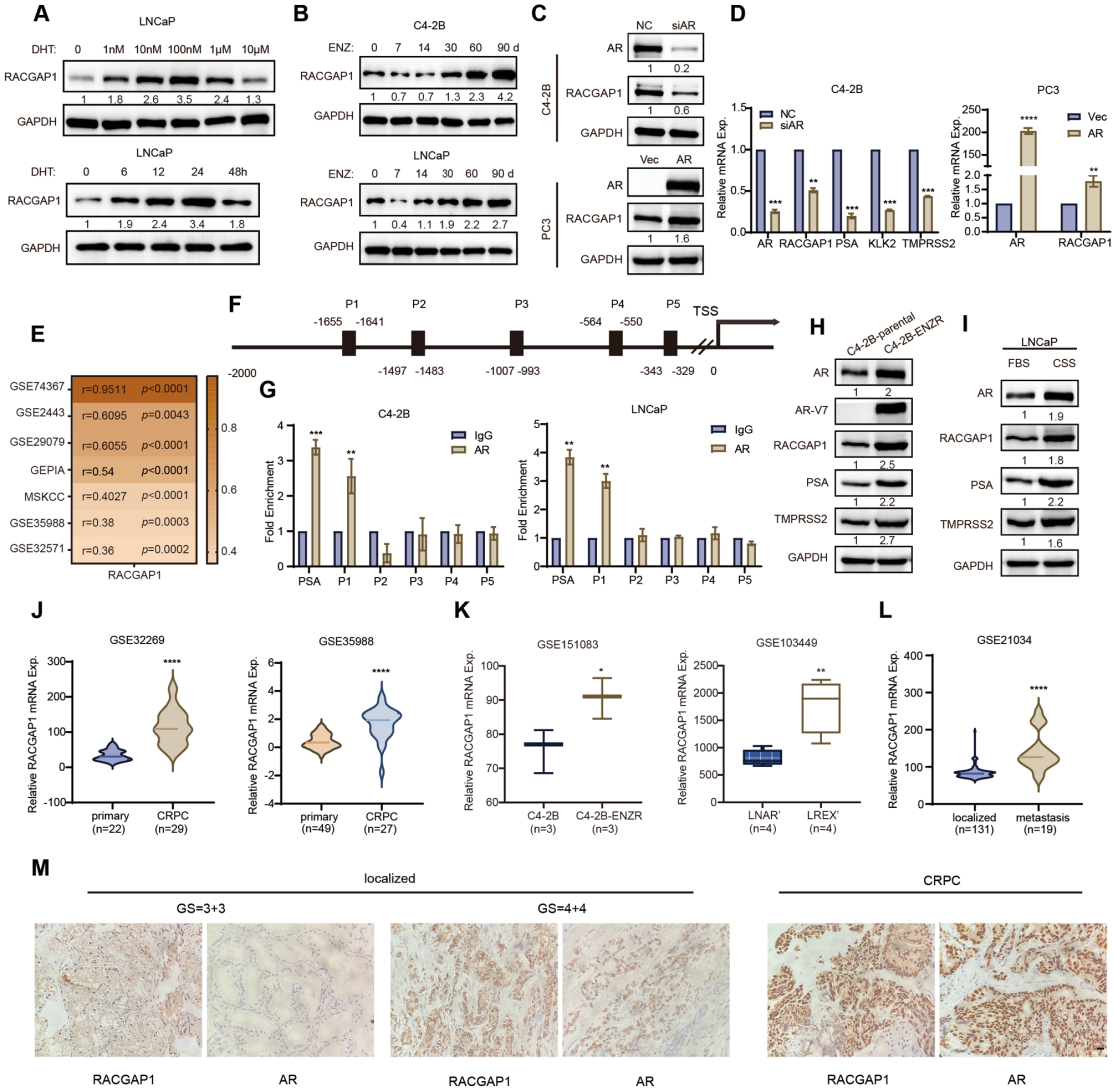
RACGAP1 is an androgen responsive gene, and positively related to AR expression in PCa. **A**, RACGAP1 protein level determined by western blotting assay in LNCaP cells after incubation with DHT. LNCaP cells were cultured in charcoal-stripped serum (CSS) medium for 3 days, and then stimulated with indicated doses of DHT for 24 h (top) or 1nM DHT for the indicated periods of time (bottom). GAPDH was used as a loading control. Densitometry analysis was performed using Image J, with target protein bands normalized to GAPDH bands. The fold change between the experimental group and the control group was calculated based on the normalized bands. h, hours. **B**, RACGAP1 protein level determined at the indicated time points by western blotting assay. LNCaP and C4-2B cells were treated with 1 µM enzalutamide for 3 months. ENZ, enzalutamide. RACGAP1 bands were normalized to GAPDH bands. The fold change between the experimental group and the control group was calculated based on the normalized bands. d, days. **C** and **D**, The protein and mRNA levels of AR, RACGAP1 and AR target genes measured in indicated PCa cells by western blotting (**C**) and qRT-PCR (**D**) assays. C4-2B cells were transfected with siRNA targeting AR, and PC3 cells were transiently transfected with AR expression plasmid. The target protein bands were normalized to GAPDH bands. The fold change between the experimental group and the control group was calculated based on the normalized bands. **E**, The heatmap representing the correlation coefficients between the mRNA levels of RACGAP1 and AR in public PCa datasets. Color represented correlation coefficients, and *p* values were displayed. **F**, The schematic diagram of five putative AR binding sites on the RACGAP1 gene promoter predicted by JASPAR (http://jaspar.genereg.net). **G**, ChIP-qPCR analysis of AR recruitment on RACGAP1 promotor region in C4-2B and LNCaP cells. Purified rabbit IgG was used as negative control. Primers for the AR binding site in PSA promoter were used as positive control. **H**, The protein levels of RACGAP1, AR/AR-V7, and their target genes determined by western blotting assays in C4-2B-Parental and C4-2B-ENZR cells. The target protein bands were normalized to GAPDH bands. The fold change between the experimental group and the control group was calculated based on the normalized bands. **I**, The protein levels of RACGAP1, AR, and AR target genes in LNCaP cells measured by western blotting. LNCaP cells were cultured with prolonged androgen deprivation for 3 months. The target protein bands were normalized to GAPDH bands. The fold change between the experimental group and the control group was calculated based on the normalized bands. FBS, fatal bovine serum. CSS, charcoal-stripped serum. **J**, RACGAP1 expression in CRPC patients compared to the primary tissues from public datasets of GSE32269 and GSE35988. **K**, RACGAP1 expression in the ENZR datasets including cell lines and patient-derived xenografts. The mRNA levels of RACGAP1 in C4-2B and C4-2B-ENZR cells (GSE151083); vehicle-treated (LNCaP/AR, LNAR’) and ENZR tumors (LREX’; GSE103449) in mouse xenograft model. **L**, Analysis of RACGAP1 expression in PCa metastasis tissues compared to localized tissues in GSE21034 (localized vs. metastasis). **M**, Representative IHC images of RACGAP1 and AR in PCa tissues. GS, Gleason score. Scale bar, 20 μm. D, G, J-L (*p < 0.05, **p < 0.01, ***p < 0.001, ****p < 0.0001)

To investigate whether RACGAP1 is regulated by the AR signaling pathway, LNCaP and C4-2B cells were treated with the AR agonist, dihydrotestosterone (DHT), or the AR inhibitor, enzalutamide, followed by interrogation of RACGAP1 expression. As shown in upper panel of Fig. 1A, RACGAP1 protein level varied depending on both the dose and duration of DHT treatment. The expression of RACGAP1 increased with treatment using low doses of DHT (0-100nM) but decreased at higher levels of DHT (above 1µM). When treating LNCaP cells with 1nM DHT for varying durations, the expression of RACGAP1 increased in the short-term exposure (0–24 h), but decreased in the long-term exposure (48 h) (Fig. 1A, lower panel). We then treated LNCaP cells with 10µM enzalutamide and observed that within 48 h of short-term treatment, there was an increase in RACGAP1 expression. When the enzalutamide treatment time expanded over 96 h, the expression of RACGAP1 decreased (Fig. S1A). During prolonged enzalutamide treatment (~ 3 months) in both LNCaP and C4-2B cells, we observed a reduction of RACGAP1 expression within 7 days, but significant augment when resistance to enzalutamide occurs (Fig. 1B). We then examined the expression of RACGAP1 in PCa cells. As shown in Fig. S1B, we found the protein expression of RACGAP1 was relatively higher in LNCaP, 22RV1 and PC3 cells compared to C4-2B cells. A significant positive correlation between the expression of RACGAP1 and AR was identified in PCa cells. AR knockdown decreased RACGAP1 expression in 22RV1 and C4-2B cells, whereas AR overexpression increased RACGAP1 expression at both mRNA and protein levels in PC3 cells (Fig. 1C-D, and Fig. S1C). This positive correlation between RACGAP1 and AR expression in PCa was further evidenced by bioinformatics analysis with publicly available PCa patient datasets (Fig. 1E) and CCLE dataset (Fig. S1D). Next, to determine whether AR directly regulates RACGAP1 transcription, we analyzed the promoter region of RACGAP1 using multiple online tools PROMO, JASPAR and GTRD. The results from the three online tools consistently suggested the presence of AR binding site in the RACGAP1 promoter (Fig. S1E). A total of five potential AR-binding sites were predicted by JASPAR (Fig. 1F) and verified in C4-2B and LNCaP cells by ChIP-qPCR assays. As depicted in Fig. 1G, AR was recruited to the P1 region of RACGAP1 promoter, indicating that AR can directly activate RACGAP1 transcription by binding to the promoter region of RACGAP1 in PCa cells. Similarly, ChIP-qPCR assays indicated that AR-V7 also bound to the P1 region of the RACGAP1 promoter in 22RV1 and C4-2B-ENZR cells (Fig. S1F). Moreover, luciferase reporter assay showed that AR activated RACGAP1 promoter activity in LNCaP cells with the treatment of low concentration of DHT (100nM, 24 h). On the contrary, AR inhibited RACGAP1 promoter activity in LNCaP cells under the treatment of 10µM DHT (Fig. S1G). As RACGAP1 was transcriptionally activated by AR, we speculated that it is differentially expressed during PCa progression. Western blotting and qRT-PCR assays revealed that the expression of RACGAP1 was upregulated in C4-2B-ENZR and long-term androgen deprived LNCaP cells, in which AR/AR-V7 pathway was abnormally activated (Fig. 1H-I and Fig. S1H). Further transcriptome analysis with publicly available datasets demonstrated high expression of RACGAP1 in CRPC (Fig. 1J and Fig. S1I-J), ENZR cell lines and xenografts (Fig. 1K and Fig. S1K-L). Compared to localized PCa, RACGAP1 was found to be upregulated in metastasis PCa (Fig. 1L and Fig. S1M). Next, to investigate the correlation between RACGAP1 and AR in clinical PCa samples, we examined RACGAP1 and AR protein levels with IHC staining using three tissue microarrays containing 132 cases of localized PCa and 25 CRPC tissue specimens. RACGAP1 protein expression was mainly localized in the cytoplasm in localized PCa samples with low Gleason score. Of note, nuclear staining of RACGAP1 was frequently seen in PCa cases with high Gleason score (Fig. 1M). In patients with localized PCa, high RACGAP1 levels were associated with high Gleason score (*p* < 0.0001), lymphatic invasion (*p* = 0.0087), biochemical recurrence (*p* = 0.017) and high tumor stages (*p* = 0.0076) (Supplementary Table S4). As shown in Fig. S1N, we observed a significant increase in RACGAP1 expression in CRPC samples compared to localized PCa samples. Furthermore, RACGAP1 was highly expressed in PCa patients with high Gleason score compared to those with low Gleason score (Fig. S1O). Of note, we observed that RACGAP1 was positively correlated with AR expression in localized PCa tissues (*r* = 0.37, *p* < 0.0001) and CRPC tissues (*r* = 0.59, *p* = 0.0021) (Fig. 1M and Fig. S1P). In all, these results suggested significant association between protein expression of RACGAP1 and AR in PCa progression.

### Nuclear RACGAP1 regulates the AR/AR-V7 signaling pathway and interacts with the NTD of AR in PCa cells

**Fig. 2:**
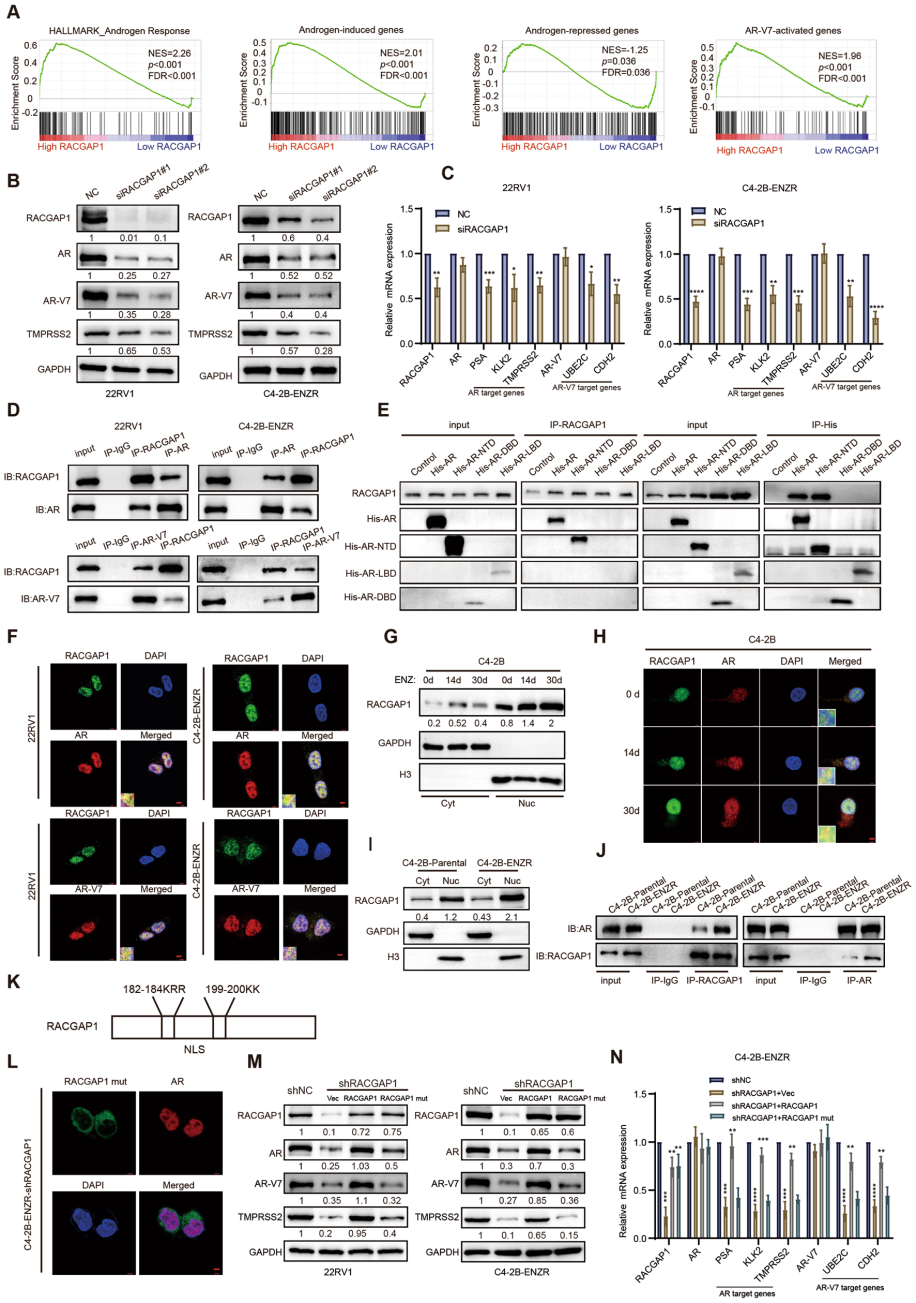
RACGAP1 regulates AR/AR-V7 protein expression by interacting with AR at the NTD. **A**, Enrichment of AR-mediated gene program analyzed by GSEA. GSEA analysis of AR related signatures with CRPC patients in GSE70768 grouped by median expression of RACGAP1. Androgen response signature was downloaded from MsigDB (https://www.gsea-msigdb.org/). The sources of signatures for androgen-induced or -repressed genes, as well as AR-V7-activated genes, were detailed in the Materials and Methods. NES, normalized enrichment score. **B**, Western blotting analysis of AR, AR-V7 and TMPRSS2 protein levels in 22RV1 and C4-2B-ENZR cells after RACGAP1 knockdown with two independent siRNA targeting RACGAP1 (siRACGAP1#1, siRACGAP1#2). The target protein bands were normalized to GAPDH bands. The fold change between the experimental group and the control group was calculated based on the normalized bands. **C**, qRT-PCR analysis of AR, AR-V7 and their target genes in 22RV1 and C4-2B-ENZR cells upon RACGAP1 knockdown, siRACGAP1#2 were utilized as siRACGAP1. **D**, The interaction of RACGAP1 and AR/AR-V7 performed in 22RV1 and C4-2B-ENZR cells by Co-IP assays. Cell lysates were immunoprecipitated with indicated antibodies. IgG was used as negative control. **E**, HEK293T cells transfected with His-AR (full length), His-AR-NTD, His-AR-DBD, His-AR-LBD plasmids or empty vector were lysed and subjected to pull-down assays using His-tag or anti-RACGAP1 antibody. Then blotted with the indicated antibodies. **F**, The localization of RACGAP1 and AR/AR-V7 protein in 22RV1 and C4-2B-ENZR cells by IF assays. Representative images were shown with a 5 μm scale-bar. RACGAP1: green; AR: red; AR-V7: red; DAPI: blue. **G-H**, Changes of nuclear/cytoplasmic expression of RACGAP1 treated with enzalutamide for 0–30 days assayed by western blotting (**G**) and IF assays (**H**). Representative images were shown with a 5 μm scale-bar. RACGAP1: green; AR: red; DAPI: blue. RACGAP1 bands were normalized to GAPDH bands (total and cytosol expression) or H3 bands (nuclear expression). **I**, Cytoplasmic and nuclear RACGAP1 protein levels analyzed in C4-2B-Parental and C4-2B-ENZR cells by western blotting assays. GAPDH and H3 were used as cytoplasmic and nuclear protein loading controls, respectively. RACGAP1 bands were normalized to GAPDH bands (total and cytosol expression) or H3 bands (nuclear expression). Cyt, cytoplasm. Nuc, nucleus. **J**, Co-IP assays of RACGAP1 and AR protein using anti-AR, or anti-RACGAP1 antibody in C4-2B-Parental and C4-2B-ENZR cells, followed by western blotting analysis with indicated antibodies. IgG was used as negative control. **K**, The schematic diagram of NLS sequence of RACGAP1. **L**, Representative IF images of RACGAP1 and AR protein localization in C4-2B-ENZR-shRACGAP1 cells transfected with NLS-mutant RACGAP1 plasmids. Representative images were shown with a 5 μm scale-bar. RACGAP1: green; AR: red; DAPI: blue. **M ** and **N**, Western blotting (**M**) and qRT-PCR (**N**) analysis of RACGAP1, AR, its target genes PSA, KLK2, TMPRSS2, and AR-V7, as well as its target genes UBE2C and CDH2 in indicated PCa cells. 22RV1 and C4-2B-ENZR cells with RACGAP1 knockdown were transfected with P-Enter (Vec), RACGAP1 or NLS-mutant RACGAP1 expression plasmids (RACGAP1 mut). The target protein bands were normalized to GAPDH bands. The fold change between the experimental group and the control group was calculated based on the normalized bands. Vec, vector. RACGAP1 mut, NLS-mutant RACGAP1. C, N (*p < 0.05, **p < 0.01, ***p < 0.001, ****p < 0.0001.)

GSEA was performed to explore the correlation between RACGAP1 expression and AR pathway activation with transcriptome data of CRPC patients and CRPC cell line, 22RV1. The results revealed that androgen response pathway genes and androgen-induced genes were significantly enriched in the RACGAP1 highly expressed groups, whereas the androgen-repressed genes were enriched in the group with lower RACGAP1 expression (Fig. 2A and Fig. S2A). Intriguingly, AR-V7-activated genes were also enriched in CRPC patients with high RACGAP1 expression (Fig. 2A). The consistency in the expression of RACGAP1 and AR-V7 was further endorsed with GSE56701, in which AR-V7 positive CRPC patients had higher RACGAP1 expression (Fig. S2B). To further assess the regulation between RACGAP1 and AR-V7, another AR-V7 positive cell line C4-2B-ENZR was included in our study. We observed a decrease in the protein levels of AR, AR-V7 and AR target gene TMPRSS2 when RACGAP1 was depleted in 22RV1 and C4-2B-ENZR cells (Fig. 2B). The downregulation of AR target genes PSA, KLK2, TMPRSS2, as well as AR-V7 target genes UBE2C and CDH2 were also evidenced by qRT-PCR at mRNA levels (Fig. 2C). Notably, the transcript levels of AR and AR-V7 remained unchanged, suggesting that RACGAP1 regulates the AR and AR-V7 expression through post-translational modifications. In addition, Gene Ontology analysis with differentially expressed genes after RACGAP1 depletion revealed a protein binding function of RACGAP1 (Fig. S2C). We assumed that RACGAP1 modulates AR/AR-V7 expression at post-translational level though protein interactions. To test this hypothesis, we first carried out Co-IP assays in 22RV1 and C4-2B-ENZR cells. The Co-IP assay confirmed that endogenous RACGAP1 coimmunoprecipitated with AR/AR-V7 protein reciprocally (Fig. 2D). AR consists of three functional domains: the NTD, DNA-binding domain (DBD), and LBD [32]. To identify the specific RACGAP1 binding region of AR protein, plasmids containing AR-truncated mutants and full-length AR were generated and transfected into 293T cells. Co-IP with AR-truncated mutants and full-length AR suggested that RACGAP1 interacts with the AR-NTD domain, which is the common domain of AR/AR-V7, rather than the DBD or LBD of AR (Fig. 2E). Additionally, immunofluorescence assays using confocal microscopy were employed to evaluate the colocalization of AR/AR-V7 protein and RACGAP1 protein in PCa cells. We observed predominate nuclear localization of RACGAP1 and AR/AR-V7 proteins in both 22RV1 and C4-2B-ENZR cells (Fig. 2F). Importantly, prolonged enzalutamide treatment induced nuclear accumulation of RACGAP1 in C4-2B cells (Fig. 2G-H). Notable exacerbation of nuclear RACGAP1 was also observed in ENZR cells compared with its parental cells (Fig. 2I). Co-IP and immunofluorescence assays corroborated our conjectures that the binding of RACGAP1 to AR was significantly increased in nucleus in C4-2B-ENZR cells compared to its parental cells (Fig. 2J and Fig. S2D). To explore the crucial role of subcellular localization in RACGAP1-AR/AR-V7 interaction, NLS sequence (182-KRR-184/199-KK-200) mutant RACGAP1 plasmid was constructed and transfected into 22RV1 and C4-2B-ENZR cells with RACGAP1 knockdown (Fig. 2K). As shown in Fig. S2E, NLS mutant RACGAP1 mainly stayed in cytoplasm and showed no colocalization with AR (Fig. 2L and Fig. S2F). We also observed that RACGAP1 overexpression increased the protein levels of AR and AR-V7, which further induced downstream gene expression, respectively. In contrast, overexpression of the NLS mutant RACGAP1 failed to affect the protein and mRNA levels of AR and AR-V7 as well as their target genes (Fig. 2M-N and Fig. S2G). Overall, the nuclear RACGAP1 regulated the AR signaling pathway by binding to the NTD of AR and AR-V7.

### RACGAP1 inhibits the ubiquitination degradation of AR/AR-V7 by disrupting the interaction between AR/AR-V7 and MDM2

**Fig. 3:**
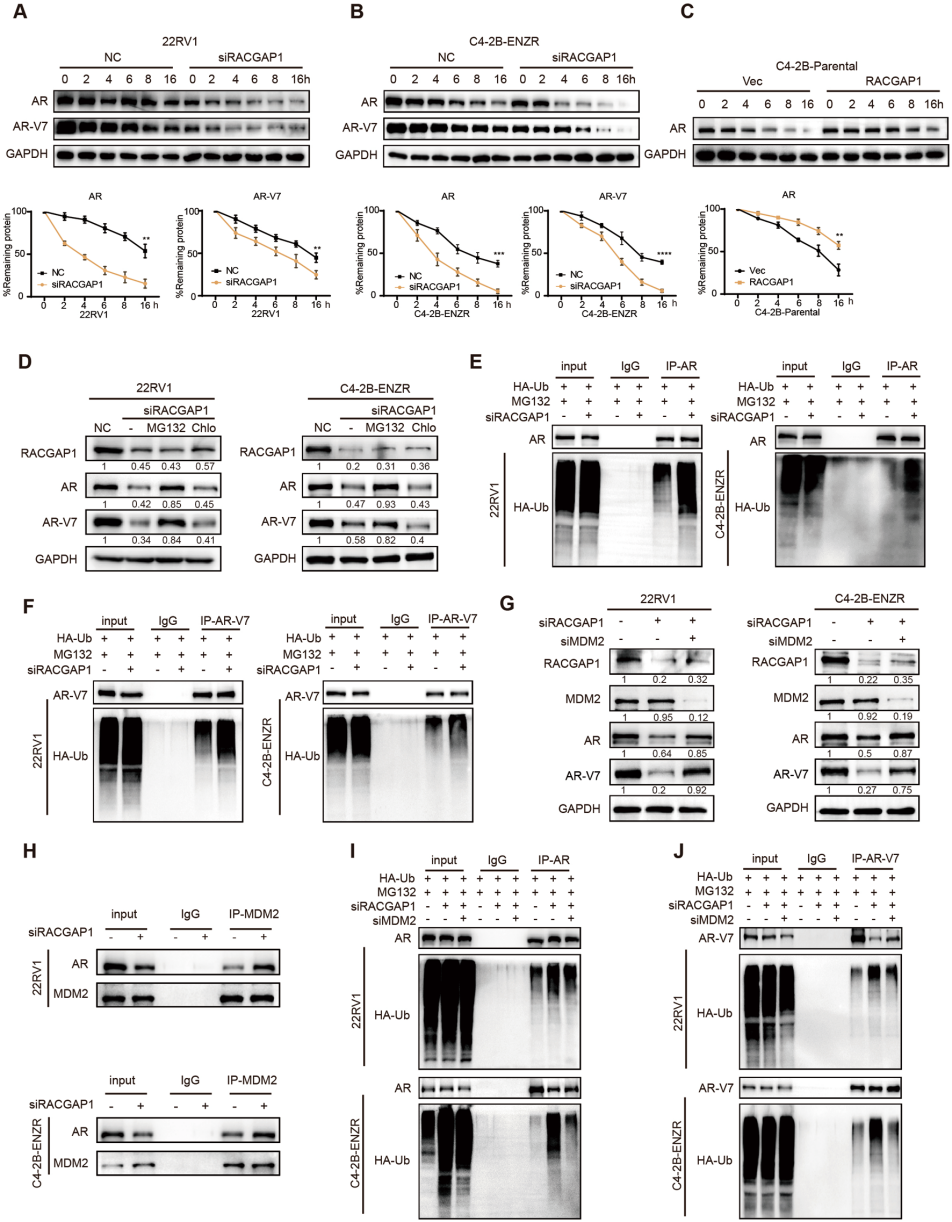
RACGAP1 inhibits the ubiquitination degradation of AR/AR-V7 by disrupting the interaction between AR/AR-V7 and MDM2. **A-C**, The protein level of AR/AR-V7 in PCa cells after incubation with cyclohexamide (CHX) for the indicated time periods determined by western blotting. C4-2B-ENZR and 22RV1 were transfected with negative control (NC) or RACGAP1 siRNA (siRACGAP1), or in C4-2B-Parental cells with empty vector (Vec) or RACGAP1 overexpression plasmids for 24 h, and then treated with 10 µg/mL CHX for 0, 2, 4, 6, 8, and 16 h. Representative images of western blotting assays (top). The densitometric quantification of AR/AR-V7 normalized to GAPDH was plotted against various time points to determine its half-life (bottom). h, hours. **D**, The protein levels of AR and AR-V7 detected by western blotting assays. 22RV1 and C4-2B-ENZR cells were transfected with NC or siRACGAP1 for 48 h and then treated with vehicle, 20 µM MG132, or 200 µM chloroquine (Chlo) for additional 24 h. The target protein bands were normalized to GAPDH bands. The fold change between the experimental group and the control group was calculated based on the normalized bands. **E **and **F**, The ubiquitination levels of AR and AR-V7 determined in PCa cells. 22RV1 and C4-2B-ENZR cells were transfected with NC or siRACGAP1 for 24 h. Cells were treated with MG132 for additional 24 h and then subjected to immunoprecipitation with AR (**E**) or AR-V7 (**F**) antibodies, followed by immunoblotting analysis with indicated antibodies. **G**, The protein levels of RACGAP1, AR, AR-V7 and MDM2 determined by western blotting assays. 22RV1 and C4-2B-ENZR cells were transfected with NC or siRACGAP1, or siRACGAP1 plus MDM2 siRNA (siMDM2). The target protein bands were normalized to GAPDH bands. The fold change between the experimental group and the control group was calculated based on the normalized bands. **H**, Co-IP assays of AR and MDM2 performed in PCa cells. 22RV1 and C4-2B-ENZR cells were transfected with NC or siRACGAP1 for 48 h, followed by western blotting analysis with indicated antibodies. **I** and **J**, The ubiquitination levels of AR and AR-V7 determined in PCa cells. 22RV1 and C4-2B-ENZR cells were transfected with NC or indicated siRNA for 24 h, then treated with MG132 for additional 24 h. Total cell lysates were collected and used for immunoprecipitation with AR (**I**) or AR-V7 (**J**) antibodies, followed by immunoblotting analysis with indicated antibodies. A-C (**p < 0.01, ***p < 0.001, ****p < 0.0001.)

To further evaluate how RACGAP1 facilitated the upregulation of AR and AR-V7 proteins, we assessed the stability of AR/AR-V7 proteins in 22RV1, C4-2B-ENZR and C4-2B-Parental cells using cycloheximide (CHX) to inhibit de novo protein synthesis. As shown in Fig. 3A-B, RACGAP1 depletion accelerated the degradation of AR/AR-V7 protein, whereas overexpressed RACGAP1 reduced the degradation rate of AR protein (Fig. 3C). In addition, the protein expression of AR/AR-V7 were rescued by proteasome inhibitor MG132 [33], but not lysosomal inhibitors chloroquine (Chlo) [34] (Fig. 3D). RACGAP1 depletion in 22RV1 and C4-2B-ENZR cells increased the ubiquitination levels of endogenous AR/AR-V7 (Fig. 3E-F). Conversely, the overexpression of RACGAP1 in C4-2B-Parental cells reduced the ubiquitination degradation of AR (Fig. S3A). These results suggested that RACGAP1 regulates AR/AR-V7 protein stability through the ubiquitin-proteasome pathway. Next, we analyzed the interactome of AR upon RACGAP1 depletion in C4-2B-ENZR cells to explore the specific mechanism underlying the AR ubiquitination degradation. Compared with the control group, an increased band at approximately 100 kDa was observed in the RACGAP1 knockdown cells, which was further evidenced to be MDM2 by western blotting assay. (Fig. S3B-C). Of note, E3 ligase MDM2 was reported to mediate the degradation of AR/AR-V7 by binding to the NTD [35]. Interfering with MDM2 simultaneously rescued the downregulation of AR/AR-V7 caused by the depletion of RACGAP1 (Fig. 3G), which confirmed that MDM2 is essential for the degradation of AR/AR-V7 induced by RACGAP1 inhibition. Co-IP assays showed that the depletion of RACGAP1 increased the protein bindings between AR and MDM2 (Fig. 3H). RACGAP1 depletion-induced AR/AR-V7 endogenous ubiquitination degradations were rescued by MDM2 knockdown (Fig. 3I-J). In addition, overexpression of MDM2 enhanced the exogenous degradation of AR/AR-V7 protein by MDM2, while RAGAP1 expression reversed MDM2-mediated exogenous ubiquitination degradation in HEK293T cells (Fig. S3D-E). In summary, these results demonstrated that RACGAP1 suppresses the MDM2-mediated ubiquitination of AR/AR-V7 by masking the binding region of the MDM2 to AR.

### Nuclear RACGAP1 confers endocrine therapy resistance of CRPC In vitro

**Fig. 4:**
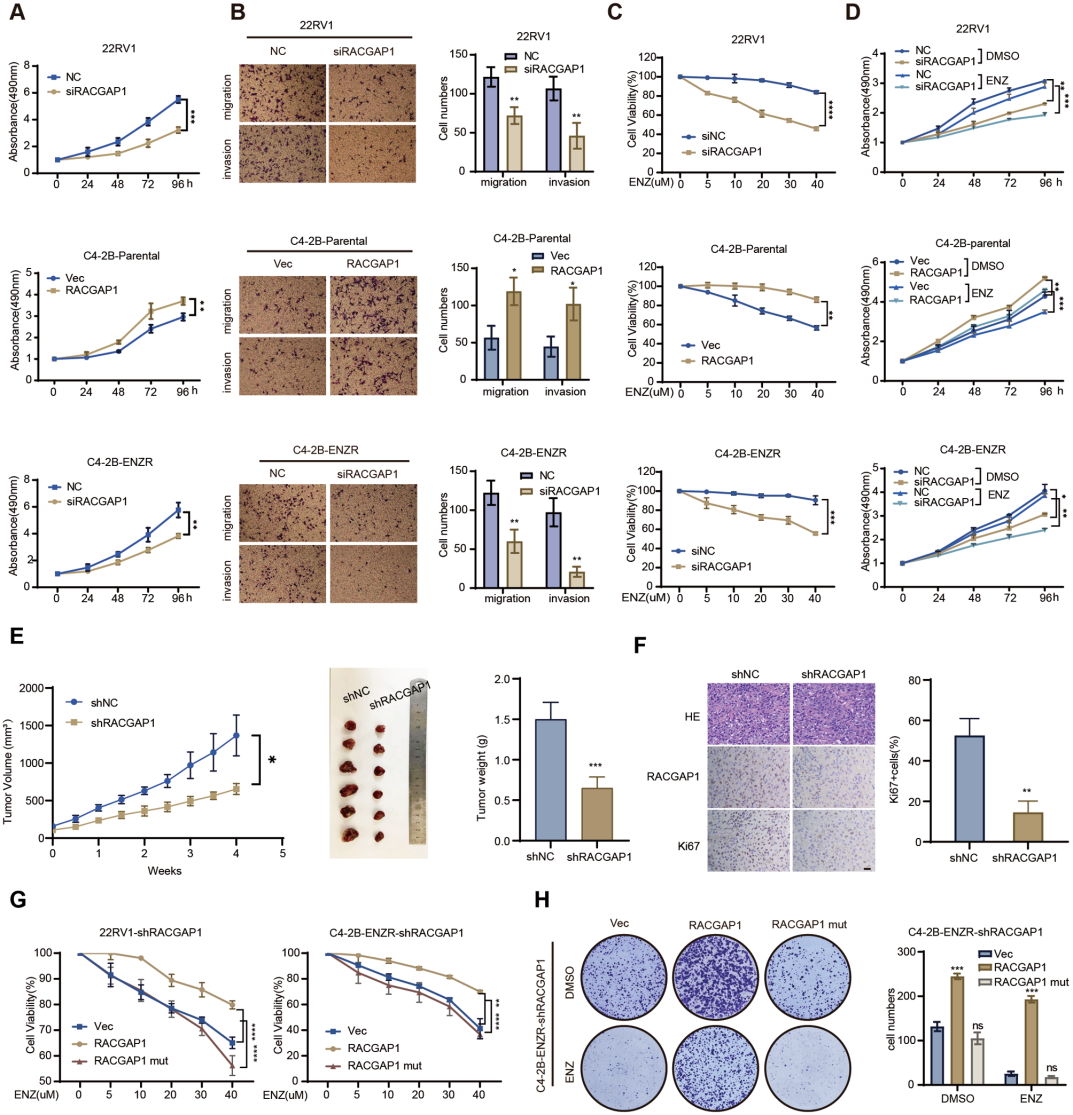
Nuclear RACGAP1 confers endocrine therapy resistance of CRPC In vitro. **A**, The cell viability in the indicated cell lines detected by MTS assays. **B**, Representative images of migration and invasion of the indicated cells. PCa cell lines were transfected as indicated and evaluated by transwell migration and matrigel invasion assays. Quantitative results of migration and invasion assays were shown in the right panel. **C**, Cell viability examined in the indicated cell lines under enzalutamide treatment by MTS assays. 22RV1, C4-2B-Parental and C4-2B-ENZR cells were transfected as indicated and treated with titrated doses of enzalutamide for 3 days. ENZ, enzalutamide. **D**, Cell proliferation determined in 22RV1, C4-2B-Parental and C4-2B-ENZR cells by MTS assays. Cells were transfected as indicated and treated with 20 µmol/L enzalutamide. ENZ, enzalutamide. h, hours. **E**, Growth curves (left) and image (middle) of xenograft tumors derived from C4-2B-ENZR cells with RACGAP1 depletion. C4-2B-ENZR-shRACGAP1 and its control cells were injected subcutaneously into nude mice (6 mice per group). Tumor size was measured twice every week. At the endpoint, tumors isolated from euthanized mice were weighed (right) and photographed. w, weeks. **F**, IHC staining of hematoxylin and eosin (H&E), RACGAP1 expression and Ki67 on tumors from each group. Representative images (left) and statistical analysis of Ki67 (right) were shown. Scale bars, 20 μm. **G**, Cell viability examined in the indicated cell lines under enzalutamide treatment by MTS assays. C4-2B-ENZR-shRACGAP1 and 22RV1-shRACGAP1 cells were transfected with Vec, RACGAP1, or RACGAP1 mut plasmids, and treated with titrated doses of enzalutamide for 3 days. ENZ, enzalutamide. **H**, Colony formation assays of indicated PCa cells with Vec, RACGAP1 or RACGAP1 mut overexpression. Quantitative analysis of colony numbers was shown in the right panel. Vec, vector. RACGAP1 mut, RACGAP1 NLS mutation. A-H (*p < 0.05, **p < 0.01, ***p < 0.001, ****p < 0.0001. ns, no significance.)

Since RACGAP1 could interact with AR to regulate the AR signaling pathway, we hypothesized that RACGAP1 promotes PCa progression and affects endocrine treatment efficacy. As shown in Fig. 4A-B and Fig. S4A, RACGAP1 depletion reduced the cellular proliferation and migration of 22RV1 and C4-2B-ENZR cells, whereas RACGAP1 overexpression increased that of C4-2B-Parental cells. Depletion of RACGAP1 in 22RV1 and C4-2B-ENZR cells improved the responsiveness of ENZR cells to enzalutamide, whereas ectopic expression of RACGAP1 considerably attenuated the sensitivity of C4-2B-Parental cells to enzalutamide (Fig. 4C). Likewise, the downregulation of RACGAP1 augmented the growth inhibition by enzalutamide in PCa ENZR cells. RACGAP1 overexpression mitigated the inhibitory effect of enzalutamide on C4-2B cells (Fig. 4D and Fig. S4B-C). Moreover, the reduction of RACGAP1 within C4-2B-ENZR xenografts in castrated nude mice resulted in a deceleration of tumors growth (Fig. 4E). The mean tumor volume at 653.3 ± 73.39 mm^3^ in RACGAP1 low expression C4-2B-ENZR xenografts but 1367 ± 273.3 mm^3^ in the control group. In the meantime, a significant decrease in average tumor weight (1.5 ± 0.21 g vs. 0.65 ± 0.14 g) and reduced cell proliferation, assessed by Ki67 IHC, were also observed in RACGAP1 knockdown xenograft (Fig. 4F and Fig. S4D). Most importantly, overexpression of RACGAP1 promoted cell proliferation and colony formation ability under enzalutamide treatment, whereas NLS mutant RACGAP1 failed to mitigate the inhibitory effects of enzalutamide on PCa cells (Fig. 4G). In addition, we observed that the pro-proliferative capacity of the RACGAP1 was abolished by the NLS mutation using cell proliferation and colony formation assays (Fig. 4H and Fig. S4E-G). These results demonstrated that nuclear RACGAP1 contributes to endocrine therapy resistance in PCa cells, and the depletion of RACGAP1 restores the efficacy of enzalutamide in CRPC in vitro.

### RACGAP1 suppression alleviates endocrine therapy resistance of CRPC In vivo

**Fig. 5:**
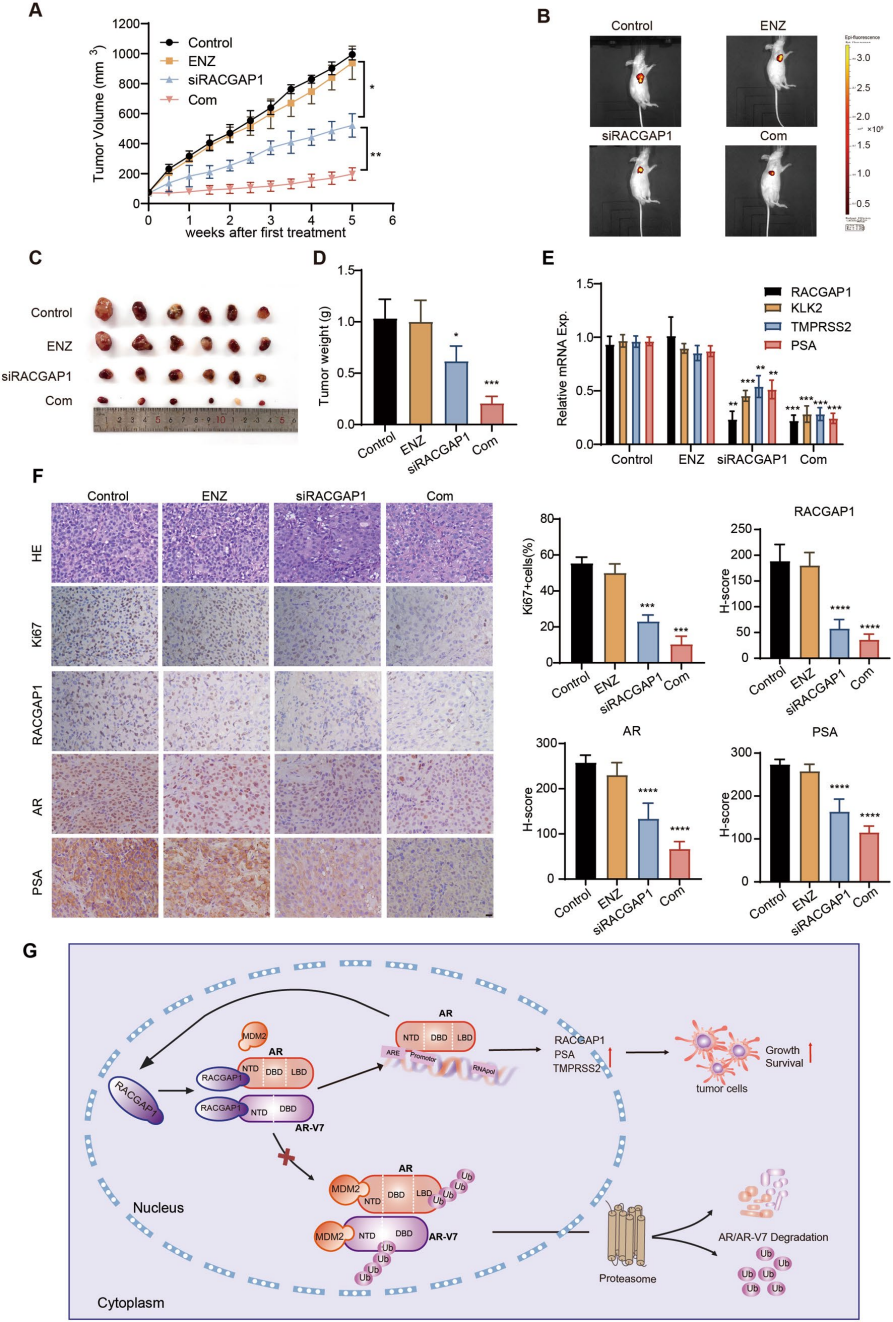
RACGAP1 suppression alleviates endocrine therapy resistance of CRPC In vivo. **A-D**, Nude mice bearing C4-2B-ENZR xenografts treated with vector control or enzalutamide (10 mg/kg p.o.), siRACGAP1(3 nmol, i.m.), or their combination for 4 weeks (*n* = 6/group). Tumor volumes were measured twice per week (**A**). Tumors were collected, photographed and weighed (**B-D**) when mice were sacrificed. w, weeks. **E**, The mRNA levels of RACGAP1, KLK2, TMPRSS2 and PSA from each group tumors measured by qRT-PCR. **F**, Representative images of Hematoxylin and eosin (H&E) staining and IHC staining of Ki67, RACGAP1, AR and PSA protein on tumor slides from each group. Scale bars, 20 μm. H-score analysis of IHC staining was shown in the right panel. **G**, A putative model illustrating the RACGAP1 and AR/AR-V7 positive feedback loop in PCa. A, D, E-F (*p < 0.05, **p < 0.01, ***p < 0.001, ****p < 0.0001.)

To further confirm whether the inhibition of RACGAP1 could alleviate endocrine therapy resistance of CRPC In vivo, we transplanted C4-2B-ENZR xenografts in castrated male nude mice. Mice were randomly divided into four groups, treated with vehicle control, enzalutamide (10 mg/kg), siRACGAP1 (3 nmol), and enzalutamide (10 mg/kg) plus siRACGAP1 (3 nmol), respectively. Treatment with enzalutamide alone led to minor decline of tumor weight, whereas the combination of siRACGAP1 and enzalutamide induced remarkable abatement in both tumor size (Control: 994.8 ± 36 mm^3^, ENZ: 939.4 ± 111.4 mm^3^, siRACGAP1: 521.7 ± 78.13 mm^3^, Com: 197.2 ± 42.16 mm^3^) and tumor weight (Control: 1.03 ± 0.19 g, ENZ: 1 ± 0.2 g, siRACGAP1: 0.62 ± 0.15 g, Com: 0.21 ± 0.07 g) (Fig. 5A-D). Additionally, we confirmed the reduction of RACGAP1 expression using In vivo cholesterol-conjugated RIG-I siRNA with qRT-PCR assays. Simultaneously, a decrease in mRNA expression of KLK2, PSA, and TMPRSS2 was observed in tumors with combined treatment (Fig. 5E). Furthermore, IHC staining revealed a robust downregulation in the protein expression of RACGAP1, AR, PSA and Ki67 when administered alone or with enzalutamide (Fig. 5F). Of note, no morphological changes of liver, kidney and others important organs were observed in all tumor groups (Fig. S5). In summary, our data showed that AR promotes RACGAP1 expression by directly binding to its promoter region, and in a reciprocal manner RACGAP1 regulates AR signaling via MDM2-dependent ubiquitination degradation of AR/AR-V7 (Fig. 5G). These results demonstrated that RACGAP1 depletion may effectively restore the endocrine therapy in CRPC, which provides a rational basis for the combined treatment for CRPC patients with RACGAP1 inhibition and enzalutamide.

## Discussion

Intensive AR signaling suppression by ADT or AR-targeting agents is the standard treatment of advanced PCa [36]. Over the past few years, novel second-generation drugs targeting the AR signaling, such as abiraterone [37] or enzalutamide [38, 39], have been proved to prolong the survival of patients with CRPC. However, the emerge of AR-V7 and aberrant activation of the AR signaling pathway confers endocrine therapy resistance, leading to inferior clinical outcome. This study shows that RACGAP1 synergistically interacts with AR through reciprocal positive feedback, leading to the hormonal treatment failure of CRPC. RACGAP1 is an androgen responsive gene and transcriptionally activated by AR. Reciprocally, nuclear accumulation of RACGAP1 stabilizes AR/AR-V7 protein and activates AR signaling pathway, further confers endocrine therapy resistance. Most importantly, inhibiting RACGAP1 in synergy with enzalutamide represents a promising therapeutic approach for advanced PCa.

RACGAP1 constitutes a significant element within the central spindle complex, actively contributing to spindle formation and stability through its interaction with KIF23 [40], thereby holding a pivotal role in cytokinesis [41]. In addition, RACGAP1 transfers to the midzone in anaphase, and accumulates to the midbody in cytokinesis [42]. RACGAP1, especially cytoplasmic accumulation of RACGPA1, promotes RhoA activation and increases microtubule dynamics, which results in cell dissemination at the late stage of mitosis [24]. Of note, it has been reported that RACGAP1 is overexpressed and associated with shorter overall survival in multiple malignances, including cancers of liver, gallbladder, breast, bladder and prostate [26, 28, 43–45]. It appears that RACGAP1 contributes to the cancer progression through both GTPase activity-dependent and independent mechanisms. In hepatocellular carcinoma, RACGAP1 promotes cell proliferation and cytokinesis in coordination with Hippo pathway through increasing activity of RhoA and polymerization of filamentous actin [26]. RACGAP1-mediated activation of RhoA can enhance the activity of YAP1/TAZ, thereby assisting PLAGL2 in promoting the progression of bladder cancer [45]. High expression of RACGAP1 in basal-like breast cancer is usually associated with poor prognosis and high recurrence rates. RACGAP1 depletion leads to proliferation inhibition and cytokinesis failure, as well as increased GTP-bound RhoA [43].

Apart from its GTPase dependent activity, several studies revealed the role of RACGAP1 as protein chaperone. In gallbladder cancer, RACGAP1 sustains LIG3 protein expression through interacting with and stabilizing LIG3 to reduce apoptosis and facilitate cells growth [28]. Yang et al. demonstrated that upregulation of RACGAP1 is positively correlated with the stage, the grade of the tumor and the biochemical recurrence, suggesting a poor outcome for patients with PCa [44]. This oncogenic function of RACGAP1 is conducted through interaction and stabilization of EZH2 [29]. In consistent with this finding, we demonstrated that RACGAP1 expression is associated with advanced clinical pathological parameters in PCa. Notably, elevated RACGAP1 expression is observed in CRPC tissues and is correlated with resistance to endocrine therapy.

RACGAP1 exhibits distinct functional roles based on its subcellular localization [46, 47]. Toshiyuki et al. [48] reported that RACGAP1 contains NLS, which can activate STAT transcription factors as a nuclear chaperone. In addition, a recent study suggested that patients with high nuclear RACGAP1 expression in colorectal cancer have worse prognosis compared to the high cytoplasmic expression of RACGAP1 [49]. In our study, we found that enzalutamide treatment of PCa cells leads to the accumulation of nuclear RACGAP1 expression, which is crucial to its interaction with AR/AR-V7. This further sustains and enhances AR signaling activity, even in the presence of enzalutamide, contributing to the development of endocrine treatment resistance. Whether trapping of RACGAP1 in the nucleus induces enzalutamide resistance or not merits further investigation. Together with these studies, we filled the gap of RACGAP1 function in interphase, while RACGAP1 was involved in central spindle complex formation and cell division in mitosis.

The ubiquitin-proteasome degradation pathway stands as a primary intracellular protein degradation mechanism, wherein the AR protein can also undergo degradation [50]. It has been reported that numerous E3 ubiquitin ligases or deubiquitinases are involved in the protein degradation process of the AR [15, 51–58]. Imbalance in AR ubiquitination dynamics, manifested as decreased ubiquitination or increased deubiquitination, intensifies AR protein levels and stimulates sequential activation of the AR pathway. Our previous study showed that KIF15 augments the interaction between USP14 and AR/AR-V7, facilitating deubiquitination and thereby contributing to ENZR of PCa [15]. In this paper, we revealed that interaction between RACGAP1 and AR/AR-V7 effectively obstructs the recruitment of MDM2, a highly characterized E3 ubiquitin ligase, impeding the ubiquitination degradation of AR/AR-V7 [53, 59]. In addition, several studies have reported the blockage of interaction between MDM2 and AR in the progression of PCa. BMI1 binding at the NTD of AR competitively inhibits MDM2 recruitment, thereby reducing proteasomal degradation and promoting stability of AR/AR-V7 protein [59]. LncRNAs, HOTAIR and LINC00675, also drive CRPC in a similar way [50, 60]. The significant role of MDM2 in AR degradation has led to the emergence of MDM2-dependent peptide-based proteolysis-targeting chimera (PROTAC) drugs that show efficacy especially in AR-V7 positive PCa [61].

Currently, research efforts are primarily directed towards exploring the potential of dual or multiple drug combinations with AR inhibitors as a strategy to overcome endocrine therapy resistance [36]. Co-targeting AR signaling and cell cycle, by combination of CDK4/6 inhibitors and ADT or AR inhibitors, are under investigation by multiple clinical trials [36, 62]. We found that RACGAP1 inhibition could re-sensitize ENZR cells to enzalutamide. Depleting RACGAP1 can have dual effects by facilitating AR protein degradation through one avenue and interfering with the mitotic functions of RACGAP1 through another mechanism. Concomitant inhibition of AR transcriptional pathway activity with enzalutamide and depletion of RACGAP1 using an in vivo siRNA system exhibited more potent suppression of PCa xenografts than either enzalutamide or siRACGAP1 alone. This combined treatment achieved dual inhibition of AR and cell mitosis, significantly inhibiting tumor growth and restoring sensitivity to enzalutamide in PCa. Furthermore, the interaction between RACGAP1 and AR/AR-V7 establishes a self-regulating positive feedback loop. RACGAP1 inhibition could break down this feedback loop, resulting in tumor suppression and re-sensitization of enzalutamide treatment. Therefore, RACGAP1 might be considered as a promising target in future combination therapies to improve PCa treatment.

In summary, our study has established that the reciprocal regulation between RACGAP1 and AR contributes to endocrine therapy failure in PCa. Importantly, the combination of RACGAP1 inhibition and enzalutamide holds promise for reversing endocrine therapy resistance in CRPC.

### Electronic supplementary material

Below is the link to the electronic supplementary material.

Supplementary Material 1

Supplementary Material 2

Supplementary Material 3

Supplementary Material 4

## Data Availability

No datasets were generated or analysed during the current study.

## Abbreviations

PCa: Prostate cancer
CRPC: Castration-resistant prostate cancer
AR: Androgen receptor
RACGAP1: Rac GTPase activating protein 1
ADT: Androgen deprivation therapy
ENZR: Enzalutamide resistance
FBS: Fetal bovine serum
DHT: Dihydrotestosterone
CSS: Charcoal-stripped fetal bovine serum
