# Supplementary material for: Reciprocal regulation between RACGAP1 and AR contributes to endocrine therapy resistance in prostate cancer
Source: Cell Commun Signal. 2024 Jun 19;22:339. doi: 10.1186/s12964-024-01703-w (PMC11186203; doi:10.1186/s12964-024-01703-w)
Supplement: Supplementary file 1 — Supplementary Material 1 [file 12964_2024_1703_MOESM1_ESM.docx]

**Supplemental Materials and Methods**

**Immunofluorescence (IF)**

Pre-treated PCa cells were seeded on glass coverslips in 24-well plates. After being allowed to adhere for 24 hours, fixed, and then incubated with primary antibodies overnight at 4℃, followed by Alexa Flour-594 or -488-conjugated secondary antibodies (Proteintech, China). The antibodies used in IF assays are RACGAP1 (NBP1-33455, NOVUS, Colorado, USA), AR (sc-7305, Santa Cruz, USA). Images were processed under Confocal Microscope (LSM980, ZEISS, Germany).

**Cell proliferation, colony formation, migration and invasion assays**

Cell viability and cell proliferation were determined by MTS assay with MTS Cell Proliferation Assay Kit (BestBio, Shanghai, China). The clonogenic ability of PCa cells was measured by plate colony formation assay. Indicated cells were plated in 6-well plate. 14 d after initial seeding, cells were fixed with 4% paraformaldehyde and stained with crystal violet solution. Colonies containing more than 50 cells were counted and plotted. The transwell assay was used to measure the migration and invasion of PCa cells using Transwell Chambers with or without coated Matrigel (Corning, NY, USA).

**Subcellular proteins extraction**

To determine the subcellular localization of RACGAP1, a nuclear/cytoplasmic fractionation kit (Beyotime, Haimen, China) was used. Cells were cultured in 10 cm dishes. After different treatments, the cytoplasmic and nuclear protein were extracted and collected with the Kit. Cytoplasmic extraction reagents were used to disrupt the cell membrane, thereby releasing intracellular components. Cell lysates were then fractionated into supernatant enriched with cytoplasmic components and pellet containing nuclear protein by high-speed centrifugation. Nuclear lysis buffer was used to extract nuclear proteins. We used GAPDH or Histone-H3 (proteintech) as controls for the cytoplasmic and nuclear fractions, respectively.

**Chromatin immunoprecipitation (CHIP) assays**

ChIP assays was conducted using ChIP Assay Kit (Millipore, Billerica, MA, USA) following the protocol of manufacturers. Indicated cells were cultured in 10 cm dishes, cross-linked with 1 % formaldehyde. The cells were washed with PBS after the cross-linking was terminated, and then added the protease inhibitor complex to the cell lysates. The products were subjected to immunoprecipitated with 5 µg of antibodies overnight at 4℃ after ultrasonic treatment. The purified associated DNA fragments were used as templates for qPCR. Primer sequences were listed in the Supplementary Table S2.

**Immunohistochemistry (IHC)**

The IHC staining was quantitatively analyzed using H-score. Briefly, each stained tissue section was assigned a numerical score based on the staining intensity (0, 1+, 2+, 3+) and the percentage of positively stained cells (0% to 100%). The H-score was calculated using the formula: H-score=intensity score × percentage of stained cells. The intensity score was assigned as follows: 0: No staining, 1+: Weak staining, 2+: Moderate staining, 3+: Strong staining. The percentage of stained cells was determined by the proportion of positively stained cells relative to the total number of cells in the tissue sample. The median H-score was used as the cutoff value. Samples with H-scores above the median were considered as high expression, and those below were considered as low expression.

**Dual Luciferase Assay**

Luciferase reporter vectors containing the RACGAP1 promoter (vector: pGL3) were constructed by Biosune Biotech (Shanghai, China). Cells were seeded in triplicate in 24-well plates and co-transfected with the Renilla expression plasmid pRL-TK and the reporter constructs for RACGAP1 promoters. Then cells were treated with different doses of DHT for 24 hours. The pRL-TK Renilla luciferase reporter vector was used as an internal control (Promega). Cell lyses were harvested for the luciferase reporter assay using the Dual‐Luciferase Reporter Assay System (Promega Corp, Madison, WI, USA) following the manufacturer’s instructions.
