# Supplementary material for: Reciprocal regulation between RACGAP1 and AR contributes to endocrine therapy resistance in prostate cancer
Source: Cell Commun Signal. 2024 Jun 19;22:339. doi: 10.1186/s12964-024-01703-w (PMC11186203; doi:10.1186/s12964-024-01703-w)
Supplement: Supplementary file 2 — Supplementary Material 2 [file 12964_2024_1703_MOESM2_ESM.docx]

**Supplementary Table S1. siRNAs used in this study.**

| **siRNA** | **Sequence** | | | |
| --- | --- | --- | --- | --- |
| siRACGAP1#1 | GCGAAGGACUUUGAGGAUUTT | | | |
| siRACGAP1#2 | GCUGAAGCAUGCACGUAAUTT | | | |
| siMDM2 | GCCAGUAUAUUAUGACUAATT | | | |
| siAR | AAGAAGGCCAGUUGUAUGGAC | | | |
| These siRNA were purchased from GenePharma (Shanghai, China).  **Supplementary Table S2. Clinicopathological demographics of 132 localized PCa patients.**   \| **Parameters** \| **Count** \| **Percentage (%)** \| \| --- \| --- \| --- \| \| **Age (years)** \|  \|  \| \| <60 \| 55 \| 42 \| \| ≥60 \| 77 \| 58 \| \| **Gleason score** \|  \|  \| \| ≤7 \| 64 \| 48 \| \| >7 \| 68 \| 52 \| \| **Lymphatic invasion** \|  \|  \| \| No \| 78 \| 59 \| \| Yes \| 54 \| 41 \| \| **T stage** \|  \|  \| \| T1、T2 \| 56 \| 42 \| \| T3、T4 \| 76 \| 58 \| \| **PSA level (ng/ml)** \|  \|  \| \| <4 \| 116 \| 88 \| \| ≥4 \| 16 \| 12 \| \| **Biochemical recurrence** \|  \|  \| \| No \| 92 \| 70 \| \| Yes \| 40 \| 30 \|   **Supplementary Table S3. Primers used in this study.**   \| **Gene** \| **Forward** \| **Reverse** \| \| --- \| --- \| --- \| \| RACGAP1 \| ATGATGCTGAATGTGCGGAAT \| CGCCAACTGGATAAATTGGACTT \| \| AR \| AAGCAGGGATGACTCTGGGA \| CTGGGTTGTCTCCTCAGTGG \| \| AR-V7 \| GAAGCTGCAAGGTCTTCTTCAA \| GGTCTGGTCATTTTGAGATGC \| \| GAPDH \| GCACCGTCAAGGCTGAGAAC \| TGGTGAAGACGCCAGTGGA \| \| PSA \| GCCTGGATCTGAGAGAGATATCATC \| ACACCTTTTTTTTTCTGGATTGTTG \| \| KLK2 \| CCATGCCTGGAGACATATCA \| TCCAGCACATGTCACTCTCC \| \| TMPRSS2 \| GGTAAACTCTCCCTGCCACA \| TACTCCAGGAAGTGGGGATG \| \| UBE2C \| TTGAACACACATGCTGCCGAG \| TGCTTTGAGTAGGTTTCTTGCAG \| \| FKBP5 \| AGAACCAAACGGAAAGGAGA \| GCCACATCTCTGCAGTCAAA \| \| **CHIP** \|  \|  \| \| PSA \| GCCTGGATCTGAGAGAGATATCATC \| ACACCTTTTTTTTTCTGGATTGTTG \| \| UBE2C \| TTGAACACACATGCTGCCGAG \| TGCTTTGAGTAGGTTTCTTGCAG \| \| P1 \| CTATATCCCCTCCTCCAAGCA \| GCCTAGGAATCACCCTTTCACA \| \| P2 \| TTGAGTGTGAAAGGGTGATTCCT \| TCTGTGACACCAGGGAACAAG \| \| P3 \| TTATGCCAAACAGGGAAGGGG \| GGCCCTATCCTATGTAGCTGT \| \| P4 \| GCTCCCAACAGTGCTTTACAC \| CCTGAGGGACTAGTGAGCCA \| \| P5 \| AATCCCAGCACTTTGGGAGG \| CGATTTTCCCACCTCAGCCT \| \| These primers were purchased from Biosune Biotechnology (Shanghai, China). \| \| \|   **Supplementary Table S4. Relation of RACGAP1 expression with clinicopathological parameters in 132 localized PCa patients.** | | | | |
| **Parameters** | | **RACGAP1 expression** | | ***p value*** |
|  |  | **Low (n=67)** | **High (n=65)** |  |
| **Age (years)** | |  |  |  |
| <60 | | 32 | 23 | 0.1493 |
| ≥60 | | 35 | 42 |  |
| **Gleason score** | |  |  |  |
| ≤7 | | 47 | 17 | <0.0001 |
| >7 | | 20 | 48 |  |
| **Lymphatic invasion** | |  |  |  |
| No | | 47 | 31 | 0.0087 |
| Yes | | 20 | 34 |  |
| **T stage** | |  |  |  |
| T1、T2 | | 36 | 20 | 0.0076 |
| T3、T4 | | 31 | 45 |  |
| **PSA level (ng/ml)** | |  |  |  |
| <4 | | 61 | 55 | 0.2578 |
| ≥4 | | 6 | 10 |  |
| **Biochemical recurrence** | |  |  |  |
| No | | 53 | 39 | 0.017 |
| Yes | | 14 | 26 |  |
|  | | | | |
