# Supplementary material for: Reciprocal regulation between RACGAP1 and AR contributes to endocrine therapy resistance in prostate cancer
Source: Cell Commun Signal. 2024 Jun 19;22:339. doi: 10.1186/s12964-024-01703-w (PMC11186203; doi:10.1186/s12964-024-01703-w)
Supplement: Supplementary file 3 — Supplementary Material 3 [file 12964_2024_1703_MOESM3_ESM.docx]

**Supplementary Figures and Legends**


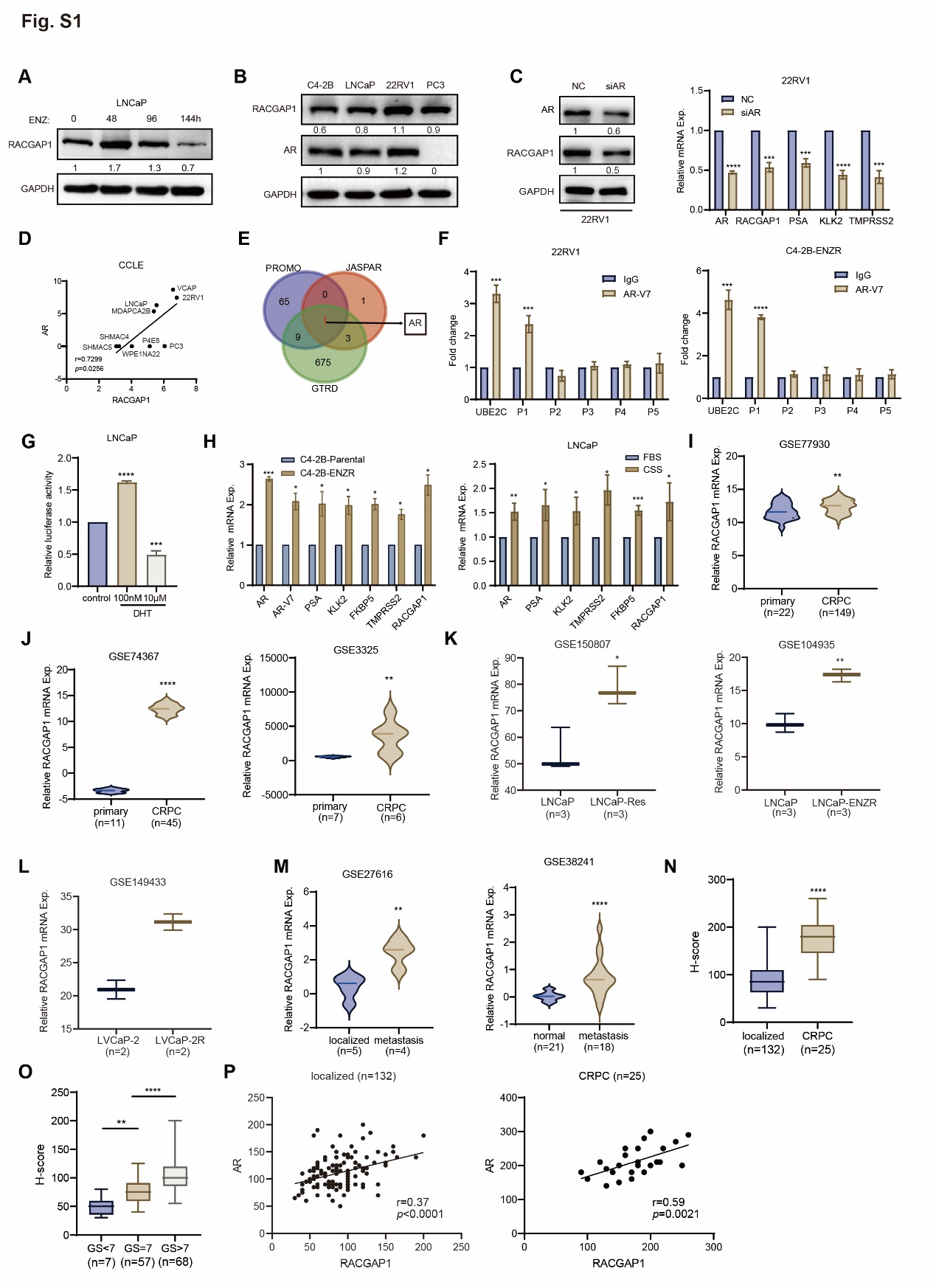
 **Supplementary Fig. 1 RACGAP1 is upregulated in advanced PCa**

**A,** RACGAP1 protein level determined by western blotting assay in LNCaP cells with treatment of 10 µM enzalutamide for the indicated periods of time. GAPDH was used as a loading control. RACGAP1 bands were normalized to GAPDH bands. The fold change between the experimental group and the control group was calculated based on the normalized bands.

**B,** The protein expression of RACGAP1 and AR in PCa cells determined by western blotting assays. The target protein bands were normalized to GAPDH bands. The fold change between the experimental group and the control group was calculated based on the normalized bands.

**C,** Western blotting (left) and qRT-PCR (right) analysis of AR, RACGAP1 and AR target genes expression in 22RV1 cells. Cells were transfected with siRNA targeting AR and blotted with indicated antibodies. The target protein bands were normalized to GAPDH bands. The fold change between the experimental group and the control group was calculated based on the normalized bands.

**D,** The correlation between the expression of RACGAP1 and AR in PCa cell lines in Cancer Cell Line Encyclopedia (CCLE) dataset at the mRNA level.

**E,** Venn diagram showing potential transcription factor of RACGAP1 predicted by PROMO (<https://alggen.lsi.upc.es/>), JASPAR (<http://jaspar.genereg.net>.), and GTRD (<https://gtrd20-06.biouml.org/>).

**F,** ChIP-qPCR analysis of AR-V7 recruitment on RACGAP1 promotor region in 22RV1 and C4-2B-ENZR cells. Purified rabbit IgG was used as negative control. Primers for the AR-V7 binding site in UBE2C promoter were used as positive control.

**G,** Luciferase reporters containing RACGAP1 promoter constructed. Luciferase reporter assays were performed in LNCaP cells transfected with RACGAP1 promoter plasmid and treated with indicated doses of DHT.

**H,** The mRNA levels of RACGAP1, AR/AR-V7, and their target genes in C4-2B-Parental and C4-2B-ENZR cells measured by qRT-PCR assays (left panel). The mRNA levels of RACGAP1, AR, and AR target genes in LNCaP cells measured by qRT-PCR assays. LNCaP cells was cultured with prolonged androgen deprivation for 3 months. FBS, fatal bovine serum. CSS, charcoal-stripped serum (right panel).

**I-J,** The mRNA expression of RACGAP1 in the CRPC tissues compared to the primary samples in GSE77930, GSE74367 and GSE3325 datasets.

**K-L,** RACGAP1 expression in the ENZR cell lines and patient-derived xenografts compared to the control groups from public datasets of GSE150807 (LNCaP vs LNCaP-resistance), GSE104935 (LNCaP vs LNCaP-ENZR) and GSE149433 (LVCaP vs LVCaP-2R). LNCaP-res, LNCaP-resistance.

**M,** Analysis of RACGAP1 expression in PCa metastasis tissues compared to localized or normal tissues in GSE27616 (localized vs metastasis) and GSE38241 (normal vs metastasis) datasets.

**N-O,** The protein expression of RACGAP1 in PCa cases at Qilu Hospital, including both localized PCa and CRPC cases (N). The protein expression of RACGAP1 in localized PCa cases with varying GS at Qilu Hospital (O). GS, Gleason scores.

**P,** The correlation between the protein expression of RACGAP1 and AR in in localized PCa tissues and CRPC samples.

C, F-I, J-K, M-O (**p < 0.05, **p < 0.01, ***p < 0.001, ****p < 0.0001.*)


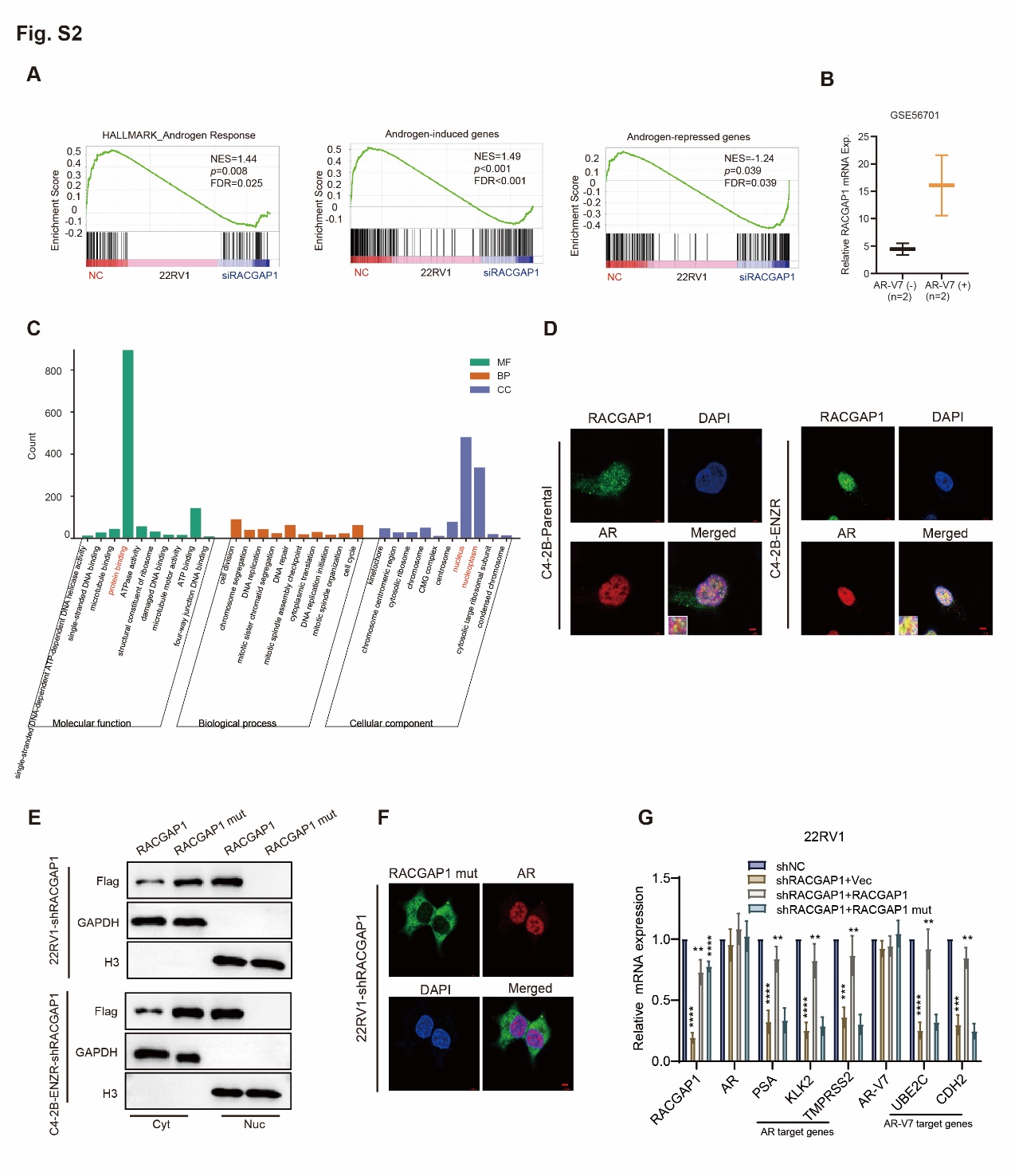


**Supplementary Fig. 2 The interaction of RACGAP1 and AR protein in nucleus.**

**A,** Enrichment of AR-mediated gene program analyzed by GSEA. 22RV1 cells were transfected with siRNA targeting RACGAP1 for 2 days. Total RNA was collected and used to perform RNA-Seq analyses. GSEA was carried out to examine the enrichment of the androgen-induced and androgen-repressed gene sets. NES, normalized enrichment score.

**B,** RACGAP1 expression in AR-V7 positive metastasis CRPC (mCRPC) patients compared to AR-V7 negative group in GSE56701 dataset.

**C,** GO enrichment analysis of differential expression genes in 22RV1 cells transfected with negative control (NC) or RACGAP1 siRNA (siRACGAP1) using online tool DAVID (<https://david.ncifcrf.gov/>).

**D,** Representative IF images of RACGAP1 and AR protein localization in C4-2B-Parental and C4-2B-ENZR cells. Representative images were shown with a 5 µm scale-bar. RACGAP1: green; AR: red; DAPI: blue.

**E,** Cytoplasmic and nuclear RACGAP1 protein distribution in PCa cells analyzed by western blotting assays. C4-2B-ENZR and 22RV1 cells with RACGAP1 knockdown were transfected with RACGAP1 or RACGAP1 NLS-mutant plasmids.

**F,** Representative IF images of RACGAP1 and AR protein localization in 22RV1-shRACGAP1 cells transfected with NLS-mutant RACGAP1 plasmid. Representative images were shown with a 5 µm scale-bar. RACGAP1: green; AR: red; DAPI: blue.

**G,** qRT-PCR analysis of RACGAP1, AR, AR-V7 and those target genes in PCa cells expressing shNC or shRACGAP1. The PCa cells expressing shRACGAP1 were transfected with Vec, RACGAP1 or NLS-mutant RACGAP1 plasmid. (**p < 0.05, **p < 0.01, ***p < 0.01, ****p < 0.001.*)


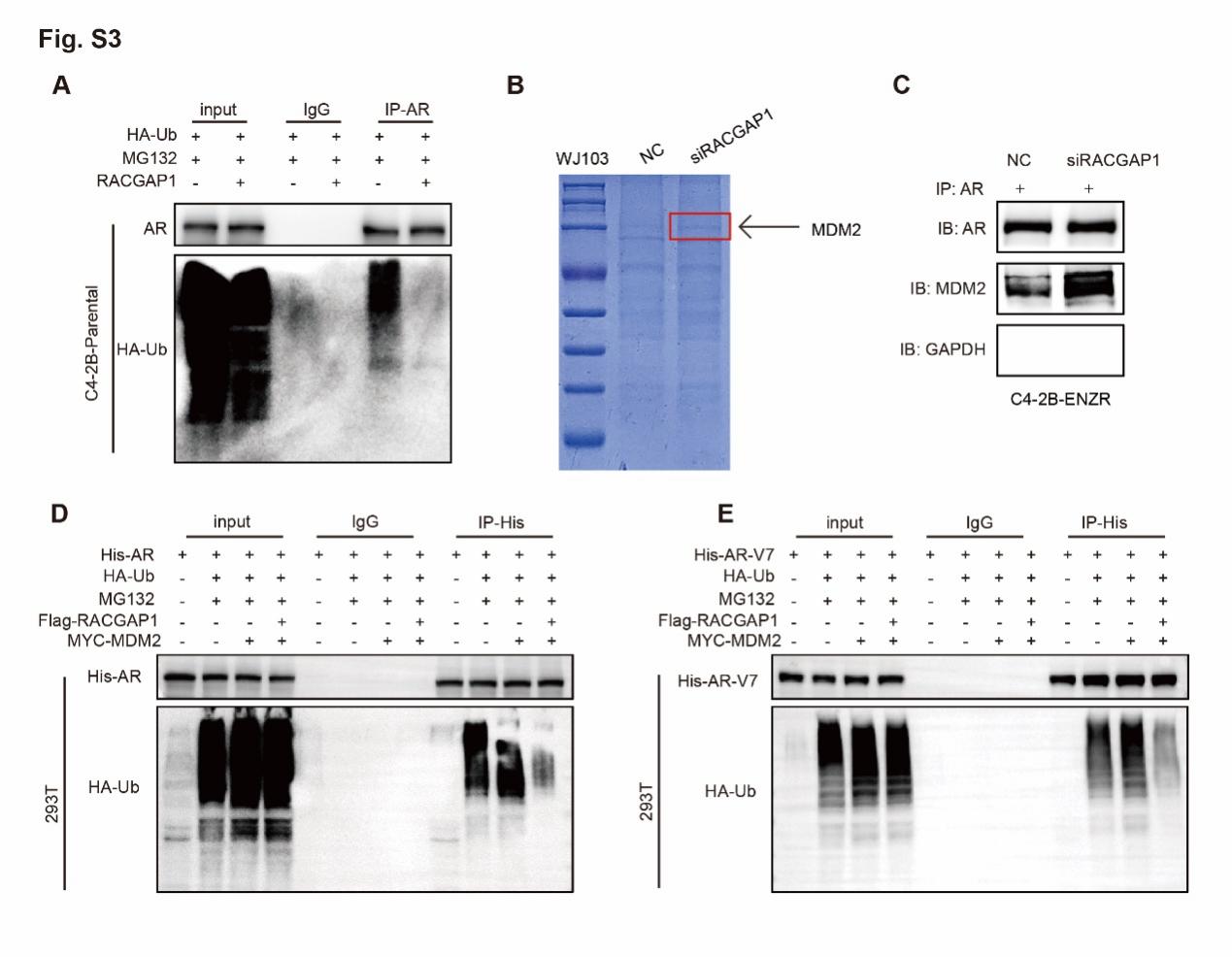


**Supplementary Fig. 3 RACGAP1 regulates ubiquitination degradation of AR protein in a MDM2-dependent manner.**

**A,** The ubiquitination levels of AR determined in PCa cells. C4-2B-Parental cells were transfected with vector or RACGAP1 plasmids for 24 hours. Cells were treated with MG132 for additional 24 hours and then subjected to immunoprecipitation with AR antibodies, followed by immunoblotting analysis with indicated antibodies.

**B,** Identification of interacting proteins with AR by Co-IP assays. C4-2B-ENZR cell was transfected with NC or siRACGAP1 for 48 hours and cell lysates were immunoprecipitated with the AR antibody. All associated proteins were separated by SDS–PAGE and Coomassie blue staining was performed.

**C,** Co-IP assays of AR interaction protein. C4-2B-ENZR cell lysates were immunoprecipitated with AR antibodies, followed by immunoblotting with the indicated antibodies.

**D,** The exogenous ubiquitination levels of AR determined in 293T cells. Cells were co-transfected with His-AR with or without Flag-RACGAP1, MYC-MDM2, and HA-Ub plasmids for 24 hours. Cells were treated with MG132 for additional 24 hours and then subjected to immunoprecipitation with HA antibodies, followed by immunoblotting analysis with indicated antibodies.

**E,** The exogenous ubiquitination levels of AR determined in 293T cells. Cells were co-transfected with His-AR-V7 with or without Flag-RACGAP1, MYC-MDM2, and HA-Ub plasmids for 24 hours. Cells were treated with MG132 for additional 24 hours and then subjected to immunoprecipitation with HA antibodies, followed by immunoblotting analysis with indicated antibodies.


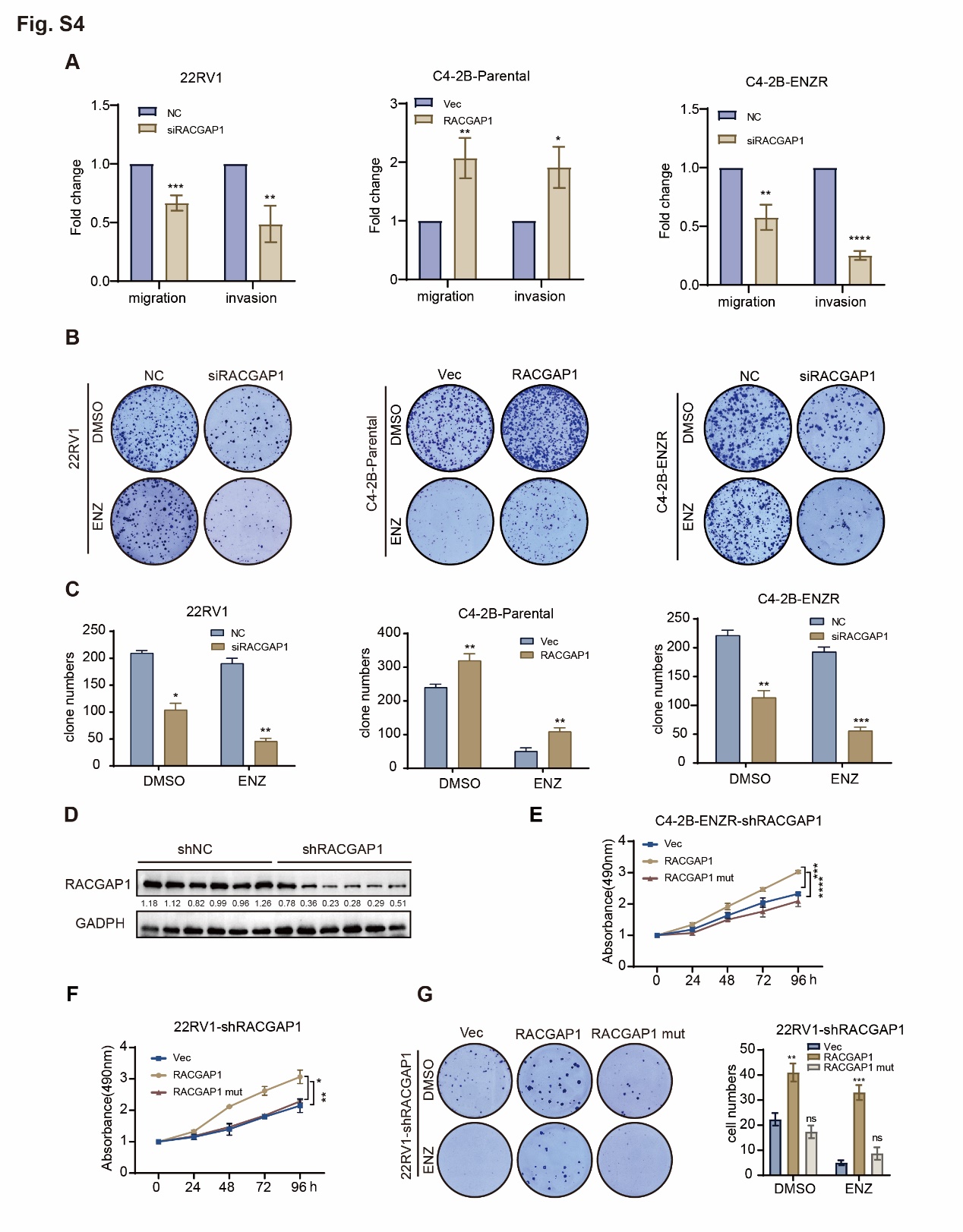


**Supplementary Fig. 4 RACGAP1 promotes cell growth in PCa.**

**A,** The quantification of invasion/migration adjusted for the differences in viability with downregulation or overexpression of RACGAP1. 22RV1, C4-2B-Parental and C4-2B-ENZR cells were transfected as indicated for 24 hours. The cell viability in the indicated cell lines detected by MTS assays.

**B-C,** Cell proliferation determined in indicated cells by colony formation assays (B). Cells were transfected as indicated and treated with 20 μmol/L enzalutamide. Quantitative analysis of colony numbers was shown in the bottom panel (C).

**D,** RACGAP1 knockdown in tumors confirmed by western blotting assay. GAPDH was used as a loading control. Densitometry analysis was performed using ImageJ, with RACGAP1 protein bands normalized to GAPDH bands.

**E-F,** Cell proliferation determined by MTS assays with 20 μmol/L enzalutamide treatment in PCa cells. C4-2B-ENZR and 22RV1 Cells with RACGAP1 knockdown were transfected with Vec, RACGAP1, or RACGAP1 mut plasmids.

**G,** Colony formation assays of indicated PCa cells with Vec, RACGAP1, or RACGAP1 mut overexpression. Quantitative analysis of colony numbers was shown in the right panel.

A, C, E-G (**p < 0.05, **p < 0.01, ***p < 0.01, ****p < 0.001.* ns, no significance.)


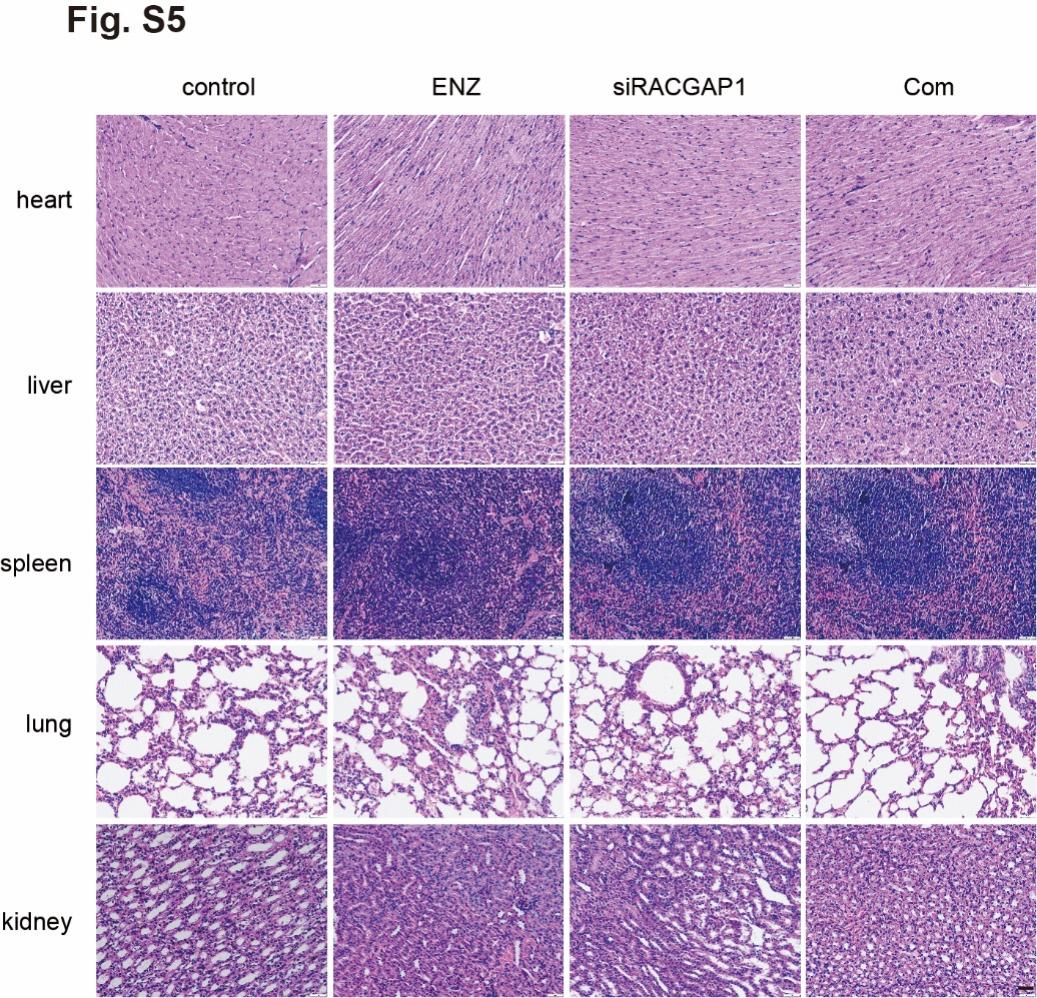


**Supplementary Fig. 5** ***In vivo* cholesterol-conjugated RIG-I siRNA drugs targeting RACGAP1 has no effect on the morphological changes in vital organs.**

**A,** Representative H&E images of heart, liver, spleen, lung and kidney tissues. In vivo cholesterol-conjugated RIG-I siRNA drugs of drug toxicity in vital organs from each group. The heart, liver, spleen, lung and kidney of the mice were stained with H&E and photographed in each group. Each group of images was magnified by a factor of 200. Representative images were shown with a 20 µm scale-bar.
