# Supplementary material for: Reciprocal regulation between RACGAP1 and AR contributes to endocrine therapy resistance in prostate cancer
Source: Cell Commun Signal. 2024 Jun 19;22:339. doi: 10.1186/s12964-024-01703-w (PMC11186203; doi:10.1186/s12964-024-01703-w)

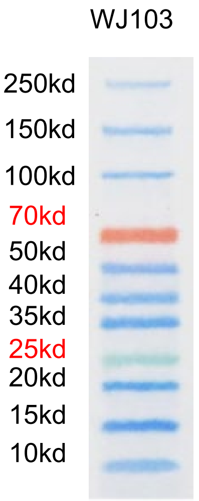

Figure 1A

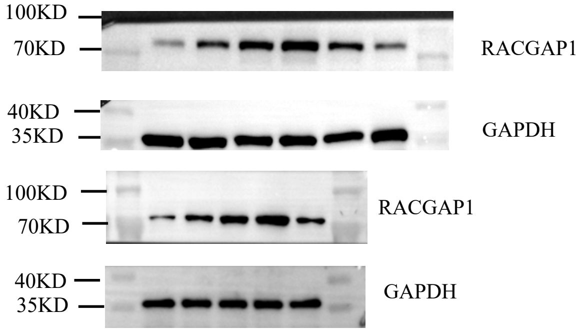

Figure 1B

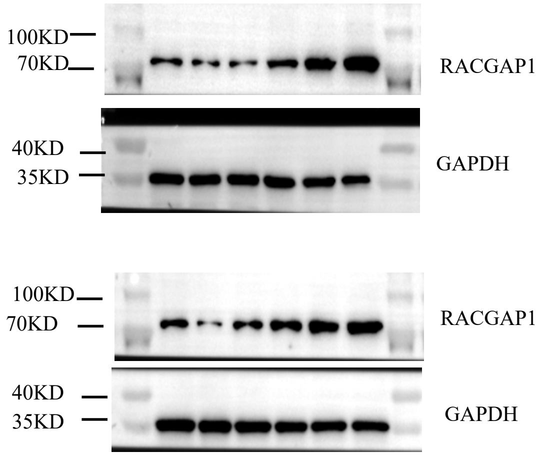

Figure 1C

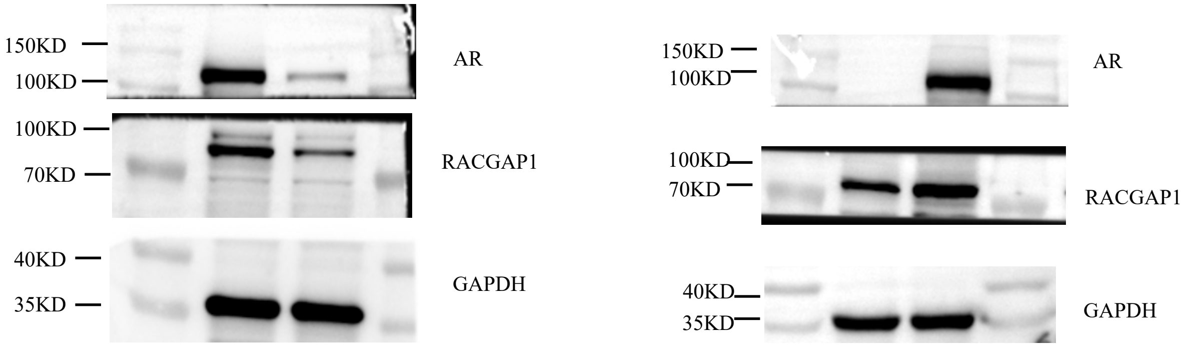

Figure 1H

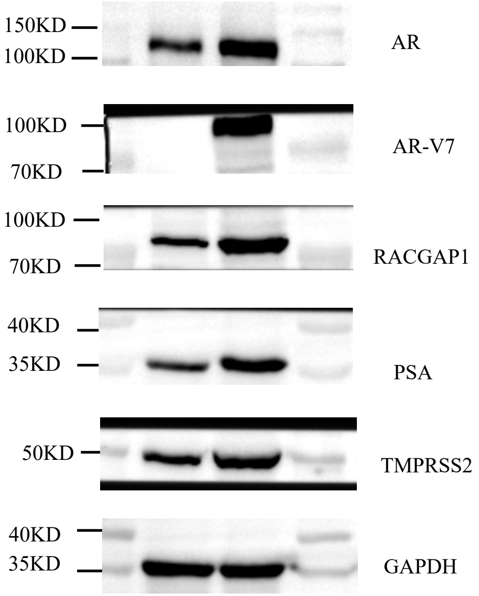

Figure 1I

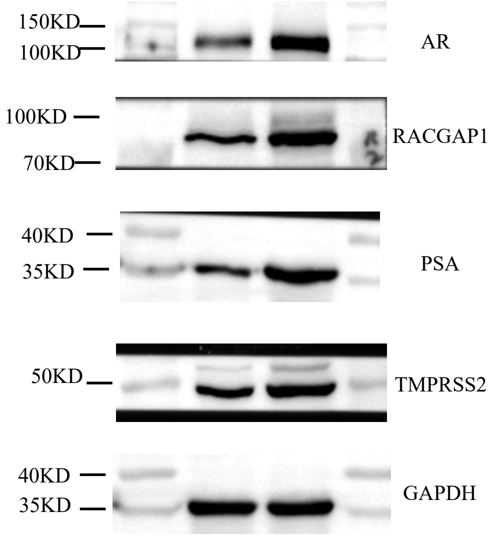

Figure 2B

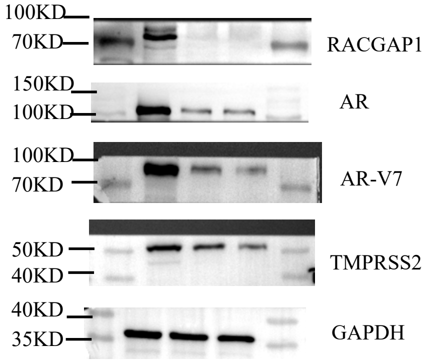

Figure 2B

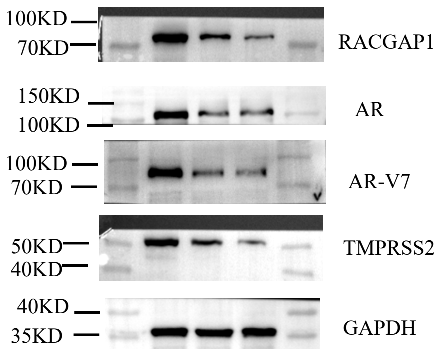

Figure 2D

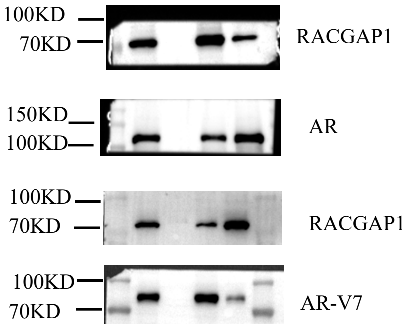

Figure 2D

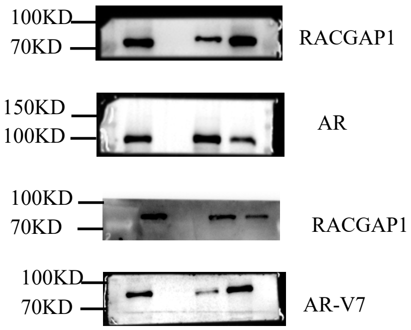

Figure 2E

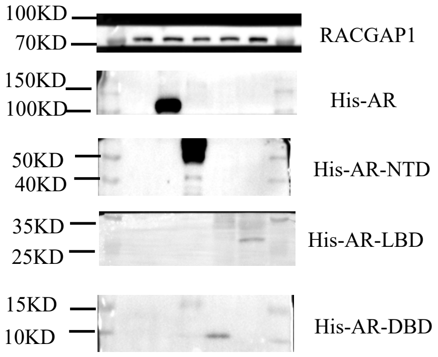

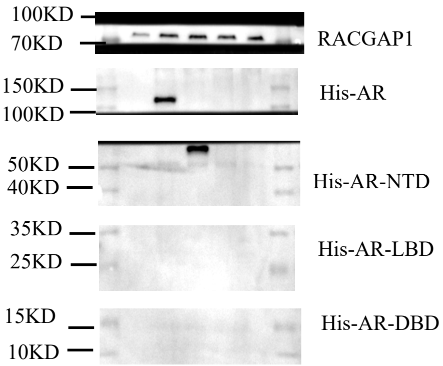

Figure 2E

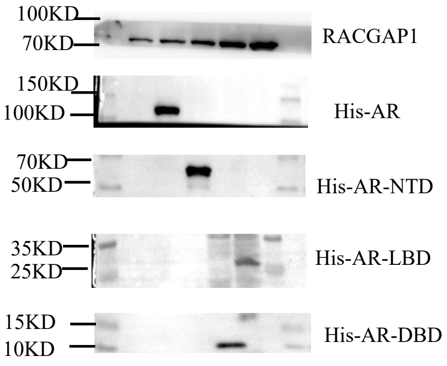

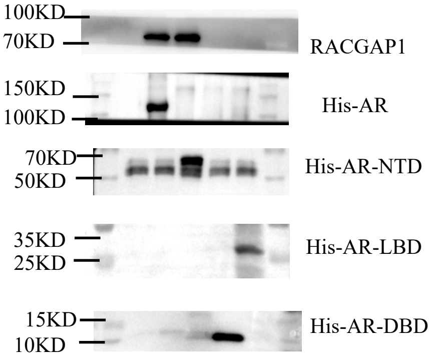

Figure 2G

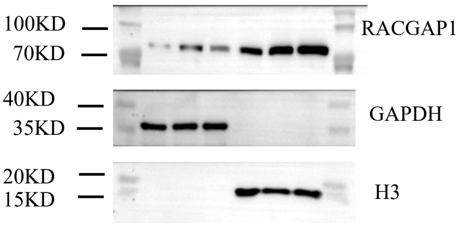

Figure 2I

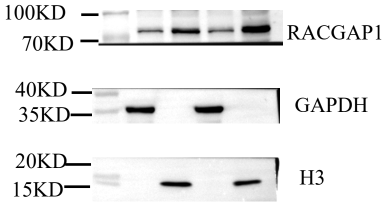

Figure 2J

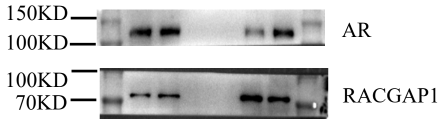

Figure 2J

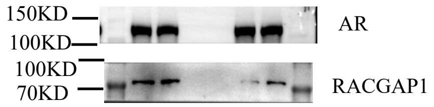

Figure 2M

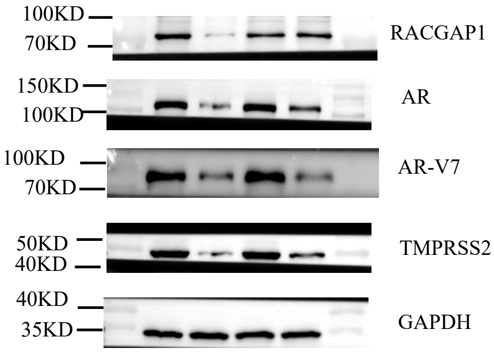

Figure 2M

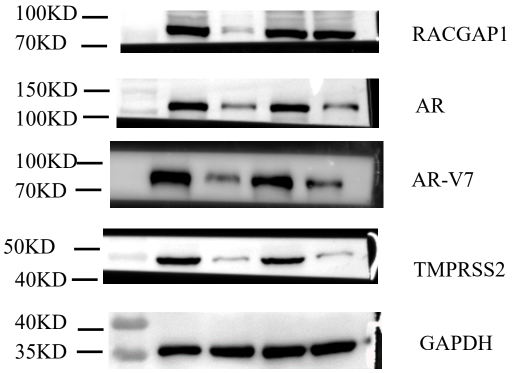

Figure 3A

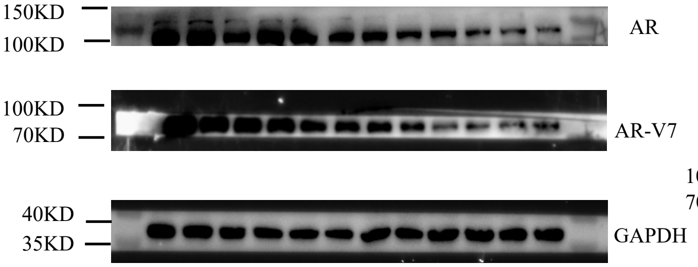

Figure 3B

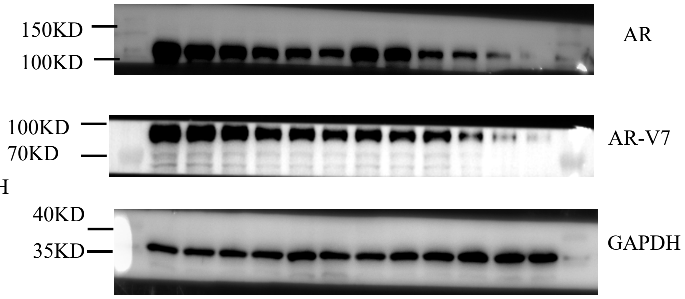

Figure 3C

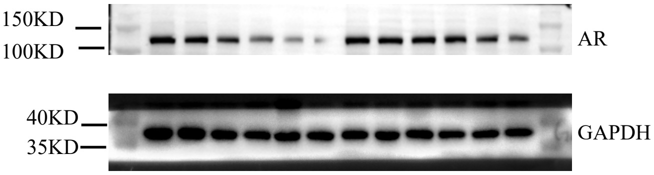

Figure 3D

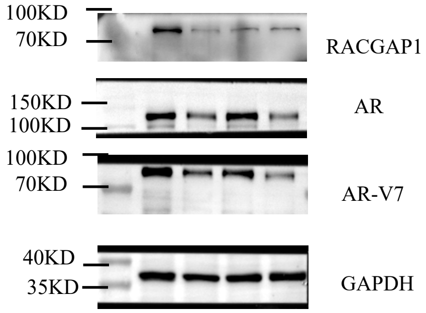

Figure 3D

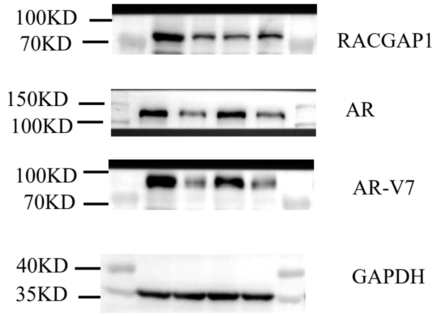

Figure 3E

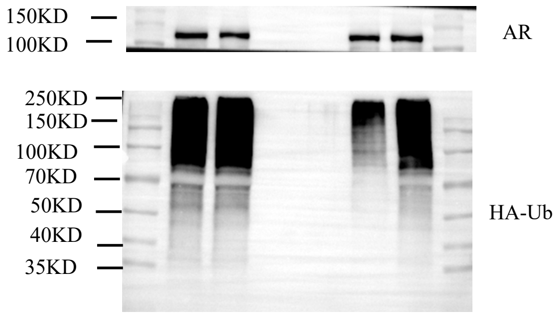

Figure 3E

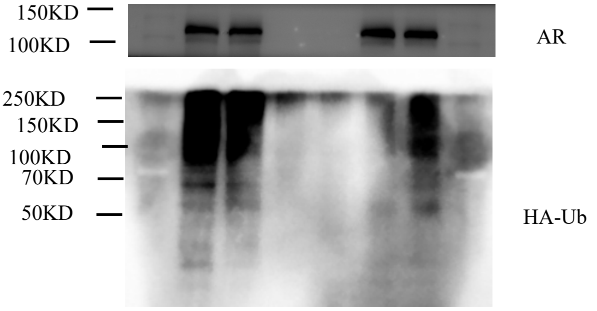

Figure 3F

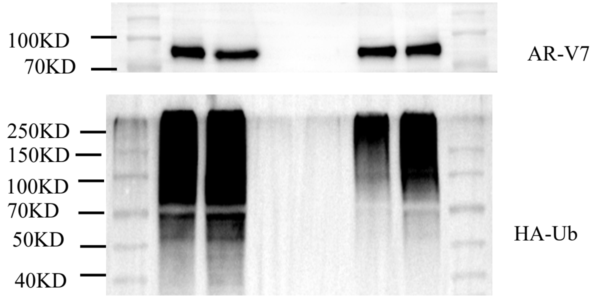

Figure 3F

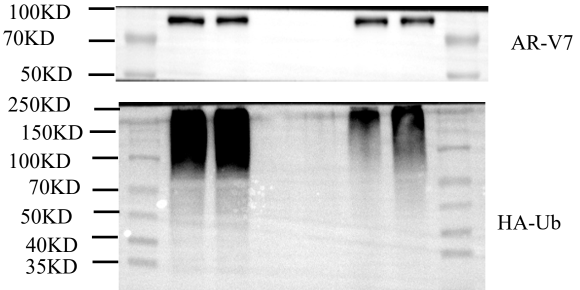

Figure 3G

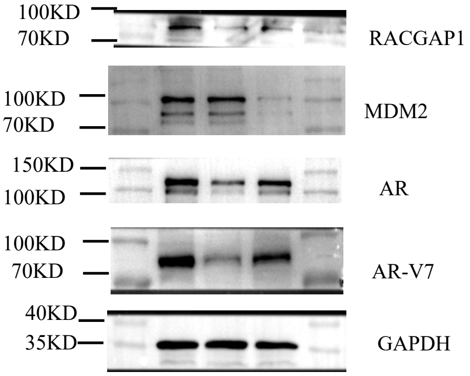

Figure 3G

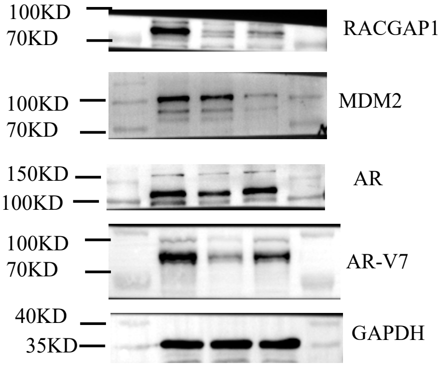

Figure 3H

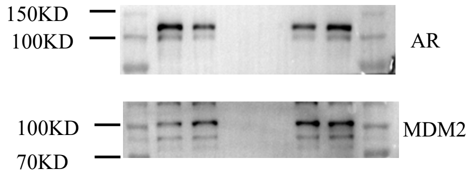

Figure 3H

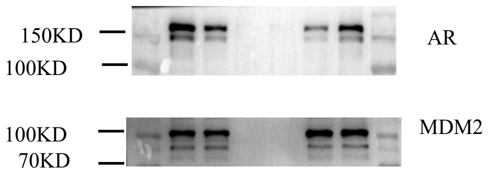

Figure 3I

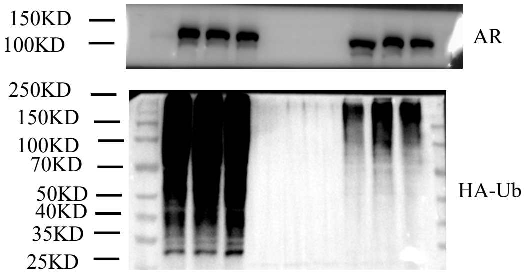

Figure 3J

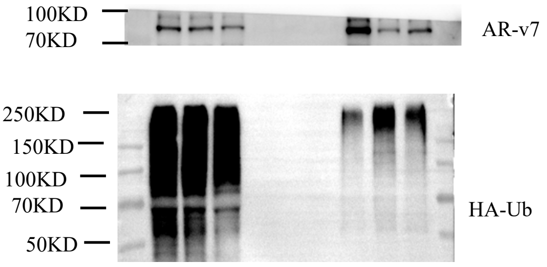

Figure 3I

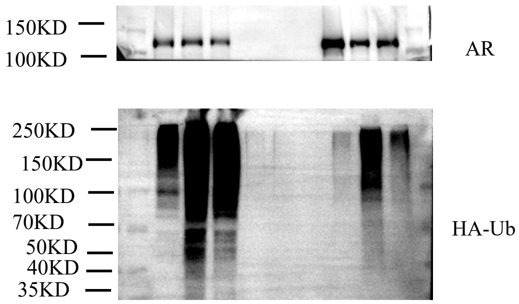

Figure 3J

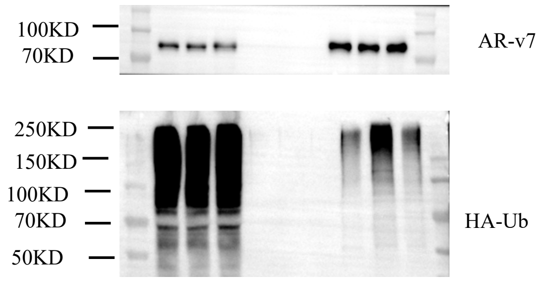

Figure S1 A

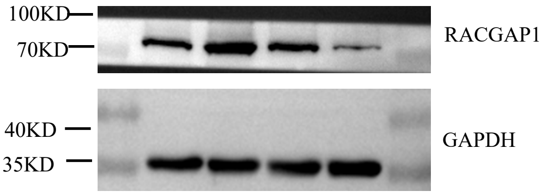

Figure S1 B

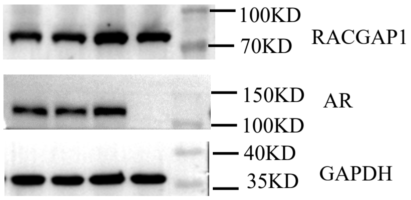

Figure S1C

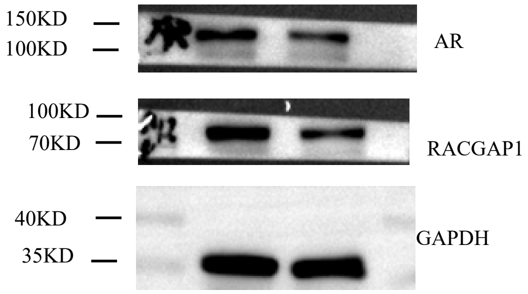

Figure S2E

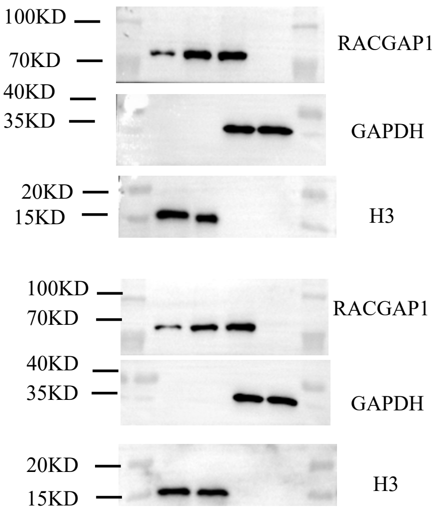

Figure S3A

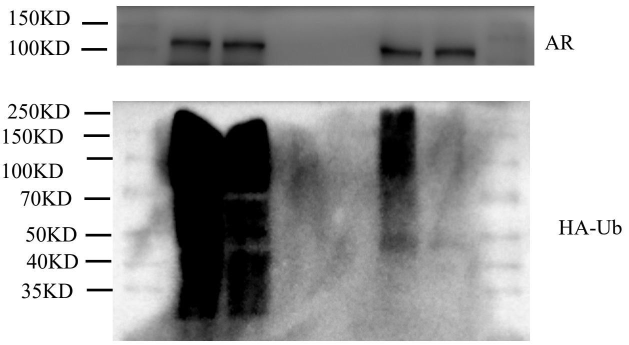

Figure S3C

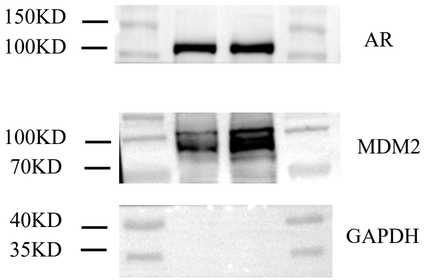

Figure S3D

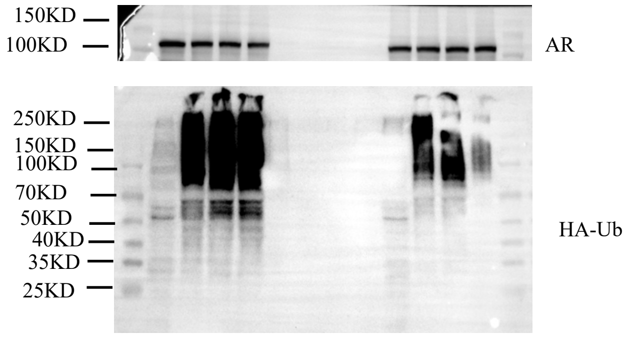

Figure S3E

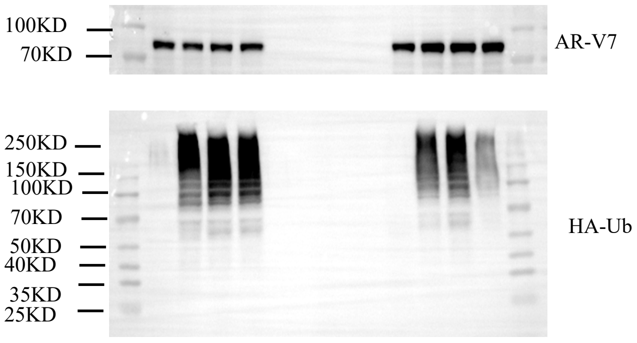

Figure S4D

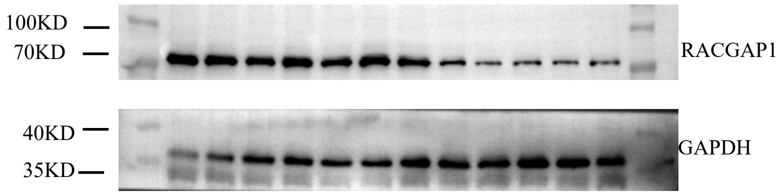

Supplement: Supplementary file 4 — Supplementary Material 4 [file 12964_2024_1703_MOESM4_ESM.pdf]
